# An increased throughput workflow to identify ion transport and membrane lysis agents for antimicrobial discovery

**DOI:** 10.1039/d5sc09781a

**Published:** 2026-03-03

**Authors:** Kylie Yang, Caleb Marsh, Lisa J. White, Fergus W. Molyneux, Thomas L. Allam, Precious I. A. Popoola, Olivia B. Keers, Matthew Rice, Kira L. F. Hilton, Hiral A. Kotak, J. Mark Sutton, Jose L. Ortega-Roldan, Charlotte K. Hind, Jennifer R. Hiscock, Cally J. E. Haynes

**Affiliations:** a Department of Chemistry, University College London 20 Gordon Street London WC1H 0AJ UK cally.haynes@ucl.ac.uk; b School of Physics, Chemistry and Earth Sciences, University of Adelaide Adelaide SA 5005 Australia; c Research and Evaluation, UKHSA Porton Down Salisbury SP4 0JG UK Charlotte.Hind@UKHSA.gov.uk; d School of Natural Sciences, University of Kent Canterbury CT2 7NH UK J.R.Hiscock@Kent.ac.uk; e School of Chemistry, University of Southampton Highfield Southampton SO17 1BJ UK

## Abstract

Small molecule ion transporters have shown promise as potential therapeutics for microbial infections, cancer and channelopathies. However, there are still gaps in our understanding of how ion transport function in model vesicle membranes translates to cell membranes of interest. The lipid composition of the membranes of bacterial and cancer cells differs markedly from normal human cells, yet the influence of the lipid composition on membrane function is rarely investigated – in part because of the low throughput and high cost of ion transport experiments. Here, we report an increased throughput Workflow to identify biologically relevant, pH-driven ion transport and membrane lysis pathways in vesicle membranes. We developed a set of four assays designed to report on different transport and lysis processes. We validated our assays against a panel of known transporters and produced a stepwise Workflow for the evaluation of libraries of compounds. We applied our Workflow to screen a library (Library 1) of 31 supramolecular, self-associating amphiphiles (SSAs) for transport and lysis activity in a range of vesicles with different lipid compositions, designed to mimic different types of cells, and consequently identified seven promising transporters. Antimicrobial experiments found that six of these promising transporters showed good antimicrobial activity against clinically relevant *Staphylococcus aureus* strains, highlighting the promise of our Workflow in identifying potential antimicrobial agents. We then applied our Workflow to investigate the ion transport and lysis properties of a second library (Library 2) of SSAs with established antimicrobial and anticancer properties, aiming to provide insight into the biological modes of action.

## Abstract 2

A new Workflow rapidly identifies biologically relevant ion transport and membrane rupture processes in vesicles of different membrane compositions. The Workflow enabled the identification of promising antimicrobials from compound libraries.

## Graphical abstract

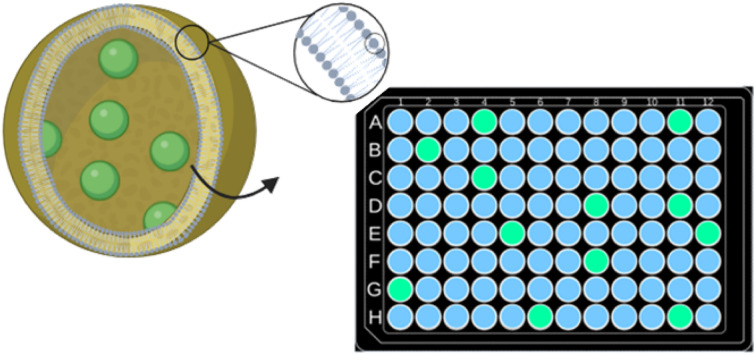

## Introduction

Small molecule ion transporters are emerging as promising therapeutics for a range of communicable and non-communicable diseases^1,2^ including channelopathies,^3–6^ microbial infections^7–17^ and cancer.^18–20^ Ion gradients, and more specifically proton gradients, are key to energy homeostasis in both bacterial cells and eukaryotic mitochondria.^21^ Synthetic ion transporters have been developed to interfere with these gradients, disrupting homeostasis which ultimately leads to cell death. This proposed mechanism of action differs fundamentally from all currently available antibiotics, presenting a novel pathway for developing new therapeutics which can bypass the widespread resistance mechanisms linked to the growing threat of antimicrobial-resistant (AMR) infections, which caused over 1.27 million deaths in 2019 alone.^22^ In 2023, AMR was implicated in roughly one-third of urinary tract infections worldwide.^23^ This growing threat to global health, which compromises the effectiveness of available life-saving treatments, underpins the ongoing need to develop novel therapeutic innovations to fight microbial infections.

Despite obvious potential, there is currently a gap in our understanding relating the design of ion transporters to biological activity.^1,24^ One reason for this could be the diversity of the target cells in which they are intended to function, including the composition of the target cell membrane(s). In practice, the mechanism and potency of synthetic ion transporters is commonly assessed using lipid vesicles as simple pre-screening models of the target cell membrane(s), that enable the identification of promising transporters, before these compounds progress into *in vitro* biological studies. The most popular choices of lipids in vesicle models contain phosphatidylcholine (PC) headgroups, which are the major component of normal human cell membranes. However, pathogenic bacteria, fungi, and cancer cells display surface lipid headgroups that are markedly different from those of normal human cells. This distinction often includes a significantly higher content of lipid headgroups such as phosphatidylglycerol (PG) and phosphatidylethanolamine (PE).^25^ There is also growing evidence that the function of synthetic ion transporters is significantly influenced by the lipid composition of vesicle membranes, including the phospholipid headgroup,^26,27^ the fatty acid region of the lipid,^28^ and the presence of sterols (such as cholesterol).^29^ This is coupled with recognition that targeting specific types of lipid may offer a route to selectively targeting particular cell types.^9^ However, transport in different lipid mixtures is not systematically explored in the field and this may contribute to the gap in our understanding of how ion transport in vesicle models relates to function in target cells.

The low throughput and high cost of ion transport experiments are key factors limiting systematic evaluation. Ideally, large libraries of transporters^30–32^ would be screened across multiple assays to determine structure–activity relationships, mechanism, and potency. However, the number of experiments becomes prohibitive when additional variables, such as different lipid compositions, are introduced. This combinatorial challenge quickly renders comprehensive studies unfeasible in terms of researcher time and overall cost. In recent work, we have sought to begin addressing the limitations of throughput in anion transport methodologies by translating the well-established lucigenin assay – which reports on Cl^−^/NO_3_^−^ antiport processes – to 96 and 384 well plate format, increasing the volume of data that can be collected simultaneously by three orders of magnitude.^33^ Busschaert and co-workers have recently developed high throughput anion antiport assays to enable the study of large compound libraries, producing data to develop a machine-learning approach to identify novel ion transport scaffolds from the DrugBank database.^30^

Building on this work, we now report the development of an increased throughput workflow based on a set of simple, cell-free assays to assess proton-coupled transport and membrane–lysis processes in synthetic vesicles. This platform was designed to rapidly identify novel ion transporters and to systematically investigate how vesicle lipid composition affects their activity and mechanism. To validate our approach, we benchmarked the outputs of our assays against a panel of known transporters. We then applied our workflow to assess the anion transport activity for a library of 31 supramolecular self-associating amphiphiles (SSAs), identifying correlations between the outcomes of our assays and antimicrobial efficacy testing, with reference to the specific lipid headgroup compositions across the vesicles and the bacterial cells. Finally, we used our workflow to assess the function of a library of five small molecules (and racemic mixtures of chiral samples) with a range of established biological activity against bacteria and ovarian cancer cells (reported elsewhere)^34^ to assess the potential impact of ion transport pathways on their known cytotoxicity profiles.

## Results and discussion

### Assay development and validation

Given the biological significance of transmembrane pH dissipation (*via* H^+^ or OH^−^ transport), we first sought to increase the throughput of the well-established HPTS (8-hydroxypyrene-1,3,6-trisulfonic acid) transport assay.^35^ Here, a membrane impermeable, pH responsive fluorescent probe is trapped inside lipid vesicles to provide a fluorescent readout on changes in intravesicular pH. The HPTS functions as an excitation ratiometric probe with a sensing range between pH 7 and 8. The acidic and basic forms of this dye have different excitation maxima (403 nm and 460 nm respectively) but both emit at 510 nm, and the ratio of emission intensities after excitation at these wavelengths correlates with the local pH.

**Fig. 1:**
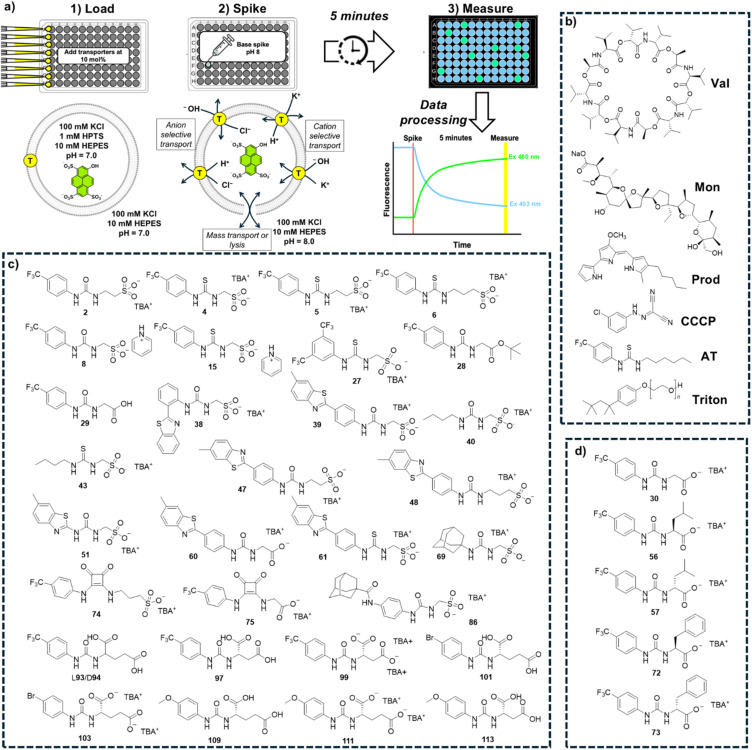
(a) An overview of our high throughput assays, performed in 96-well plate format, and cartoons showing the steps of the “catch all” KCl assay, including the possible transport or lysis processes that could give a positive result; (b) the library of known transporters (Controls) used for benchmarking and as controls throughout; (c) the 31 compounds assessed as Library 1; (d) the 5 compounds assessed as Library 2. Chiral compounds in Library 2 were also investigated as 50 : 50 racemic mixtures.

Our first iteration of this assay was designed to be a “catch all” screen to rapidly identify any transporters that enable pH dissipation across the vesicle bilayer through a variety of mechanisms. We chose to implement K^+^ and Cl^−^ ion-based assays due to the biological prevalence of these ions (in both human and bacterial cells),^36–38^ and the literature precedent that links the transport of these species to antimicrobial and anticancer outcomes.^1,39–42^ While both K^+^ and Na^+^ are naturally abundant, K^+^ is generally associated with a lower dehydration penalty than Na^+^. We therefore reasoned that K^+^ based assays could provide a more sensitive initial readout of cation transport activity. We prepared synthetic unilamellar vesicles (diameter 200 nm) containing HPTS (1 mM) and suspended in/containing KCl (100 mM) buffered to pH 7.0 using HEPES (10 mM). A transporter is added to the vesicles followed by a base pulse to raise the external pH to 8.0 and establish a pH gradient. Changes to the HPTS ratiometric fluorescence emission after adding the base pulse signals transporter-mediated pH dissipation. We adapted this assay to a 96-well plate format, manually loading vesicles and transporters into the plates using a multi-channel pipette (see SI Section S2). The plates were transferred to a fluorescence plate reader, and the base spike was delivered using an autoinjector. An “end point” ratiometric fluorescence reading was collected from each well 5 minutes after the addition of the base spike. Each plate contained both positive and negative controls to calibrate 0% and 100% pH dissipation. We designated this assay as a “catch all” assay because there are a range of distinct transport processes that could enable pH dissipation and therefore yield a positive response. This could include an anion transport process (H^+^/Cl^−^ symport or Cl^−^/OH^−^ antiport), a cation transport process (K^+^/H^+^ antiport or K^+^/OH^−^ symport), non-selective mass transport events or vesicle lysis. An overview of the “catch all” KCl assay and cartoons illustrating the potential transport mechanisms are shown in Fig. 1a.

We next designed iterations of this assay aiming to provide mechanistic information relating to any positive results we identified in the “catch all” KCl assay. By systematically substituting the anion and the cation in our buffer for more hydrophilic (and hence harder to transport) ions, we can assess the influence of the anion and the cation present on the transport efficiency. In the NMDG–Cl assay, the K^+^ cations are replaced with NMDG^+^ (*N*-methyl-d-glucamine), and we expect any cation selective transport processes to be reduced.^35^ Likewise, in the KGlu assay the Cl^−^ anions are replaced with gluconate,^43^ and we expect any anion selective transport processes to be reduced.^44^ Finally, we considered whether a positive response in our KCl assay could be the result of membrane lysis rather than a selective transport process. To enable this, we included an SRB release assay.^45^ Here we encapsulate sulforhodamine B (SRB), a membrane impermeable, self-quenching fluorescent dye (50 mM) into the vesicles. These vesicles are subsequently suspended in KCl buffer. At this concentration, SRB self-quenches and hence the fluorescence emission is low, but the addition of a membrane rupturing agent or the formation of large pore-like structures within the phospholipid bilayer releases and subsequently dilutes the SRB, causing an increase in fluorescence. We chose an SRB release assay rather than similar assays using calcein or carboxyfluorescein due to the greater pH tolerance of rhodamine fluorophores compared to fluoresceins, in the hope that this choice would afford us greater flexibility in our experimental parameters in the future. Experimental details of these assays can be found in the SI Section S2.

In summary, we developed 4 increased throughput assays:

(1) The “catch all” KCl assay (total transport).

(2) NMDG–Cl assay (reduces cation-driven transport).

(3) KGlu assay (reduces anion-driven transport).

(4) SRB assay (vesicle leakage/lysis).

To validate our assays and establish a Workflow, we then sought to benchmark the outputs of our HPTS assays against a panel of known Control ionophores in POPC (1-palmitoyl-2-oleoyl-*glycero*-3-phosphocholine) vesicles. The structures of all Control ionophores are shown in Fig. 1b. We tested single concentrations of Val (valinomycin, a K^+^ uniporter or electrogenic transporter), Mon (monensin, an electroneutral K^+^ transporter), Prod (prodigiosin, an electroneutral anion transporter) and CCCP (a H^+^ uniporter or electrogenic transporter). We also tested a combination of Val and CCCP to assess the potential synergistic combination of two complimentary uniport processes. Finally, we included AT (short named “Anion Transporter” in our laboratory) in our panel, an established anion antiporter with known biological activity^46^ that we commonly use as a control compound in our laboratory due to its ease of synthesis.^33,47^ We also introduced two (related) metrics of ion selectivity, selectivity constants *S*_anion_ and *S*_cation_ defined in eqn (1) and (2):1
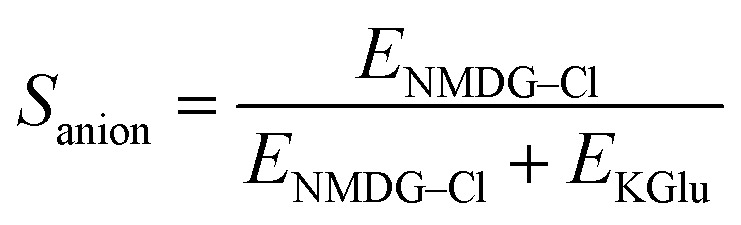
2
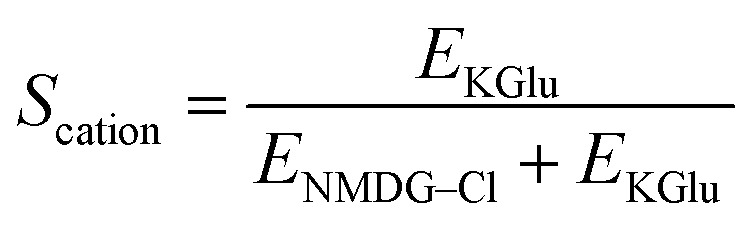
where *E*_NMDG–Cl_ is the % efflux in the NMDG–Cl assay and *E*_KGlu_ is the % efflux in the KGlu assay. % Efflux values > 100% are approximated to 100%, and % efflux < 0% are approximated to 0% (which are the physical boundaries of the assay).

Here, 0.5 < *S*_anion_ ≤ 1 implies anion selectivity, 0 ≤ *S*_anion_ < 0.5 implies cation selectivity and *S*_anion_ = 0.5 implies no selectivity. Similarly, 0.5 < *S*_cation_ ≤ 1 implies cation selectivity and 0 ≤ *S*_cation_ < 0.5 implies anion selectivity.

The results of our benchmarking experiments are summarised in Table 1. Mon showed very effective transport in the KCl and KGlu assays, with reduced transport in the NMDG–Cl assay. This indicates an expected cation selective transport process, illustrated by *S*_cation_ = 1. Prod showed a strong response in the KCl assay and the NMDG–Cl assay, but reduced function in the KGlu assay. This indicates the expected anion selective transport process, illustrated by *S*_anion_ = 0.85. The response of AT in these assays was similar to Prod, with anion selective transport observed (*S*_anion_ = 0.83). While these two anion transporters did exhibit anion selectivity (as expected), we note that the selectivity is not perfect and the observed transport in the KGlu assay was non-zero. This may be attributable to low levels of non-selective transport, lysis or a fatty acid flip flop mechanism which involves the transport of carboxylate fatty acids, then subsequent protonation.^48,49^ Since neither Prod nor AT contain an apparent binding site for cations, we believe that a selective cation transport pathway is implausible. However, we have experimentally observed that AT can mediate some degree of lysis at high concentrations, and this appears to be lipid-specific (see SI Fig. S18). Gale and co-workers have also documented the influence of fatty acids in commercial lipids on rates of pH dissipation in the presence of anion transporters,^49^ and we acknowledge that variability in the composition of commercial lipids (both between suppliers and between batches from the same supplier) could influence the apparent selectivities we observe. We also note that the selectivity factors may have a kinetic component if the transport of one species reached 100% before the end point of the experiment (5 minutes), in which case an end-point ratio can underestimate the true selectivity compared with an initial-rate analysis. While our assays do not currently enable the collection of kinetic data in multi-well format, this could be further explored by studying the initial rate of transport using lower throughput approaches.

**Table 1:** The benchmarking responses of known ionophores to the KCl, KGlu and NMDG–Cl assays in POPC vesicles. Values represent the change in the fluorescence ratio (%) normalised with respect to DMSO (0%) and a known active transporter (100%). The known transporter was chosen to be Val + CCCP in buffers that contain potassium, or Prod in buffers that contain chloride. Ionophores which mediated 100% pH dissipation under particular buffer conditions are highlighted in green. Errors are the standard deviation of *n* ≥ 3 repeats

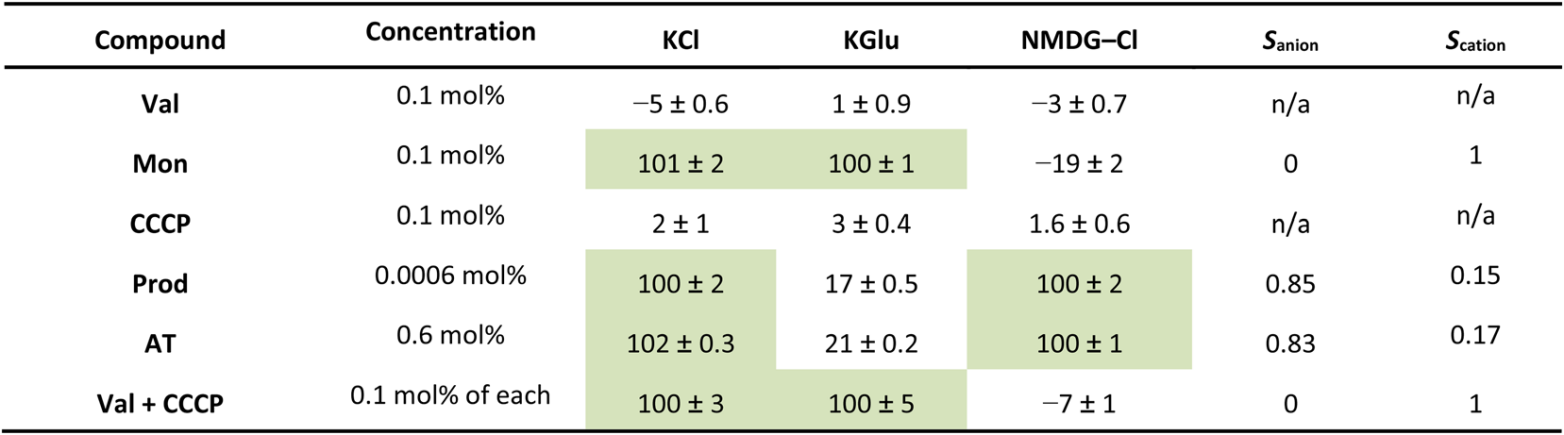

Val and CCCP, as uniporters, did not mediate significant transport in any of the assays alone. However, when combined they facilitated very effective transport in the KCl and KGlu assays, which both contain K^+^ ions, but reduced transport in the NMDG–Cl assay. This indicates an expected cation selective transport process, as represented by *S*_cation_ = 1. We attribute this to K^+^ transport by Val coupled to H^+^ transport by CCCP. Finally, we confirmed that the addition of Triton (a detergent) also consistently gave a 100% response in all assays due to assumed total lysis of the vesicles.

In summary, we established that our panel of assays could identify expected cation and anion selective transport processes mediated by established ionophores, and we therefore proceeded to use these assays as the basis for a Workflow to evaluate novel compounds.

### Ion transport and membrane lysis Workflow (termed herein “Workflow”)

**Fig. 2:**
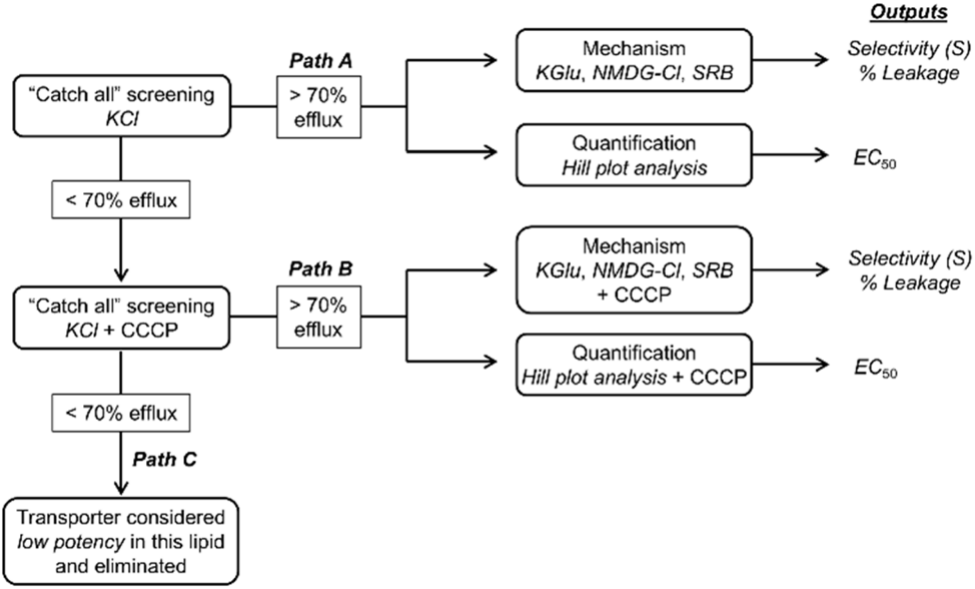
Our ion transport and leakage Workflow, designed to rapidly identify promising transporters and provide mechanistic understanding and quantification of transport. This Workflow can be followed in vesicles of different compositions.

Considering the assays at hand and the outputs of our benchmarking experiments, we propose the screening and evaluation Workflow shown in Fig. 2. The aim of this Workflow is to initially identify active transporters from a library; promising ion transport candidates are then further evaluated in assays designed to provide mechanistic understanding and quantification of transport. All transporters are screened initially in our “catch all” KCl assay at a single, high concentration (10 mol% w.r.t. lipid) in vesicles of a given lipid composition. Compounds which mediate significant transport (defined here as mediating > 70% efflux) at this concentration are considered active, electroneutral transporters, and progress *via* Path A to mechanistic assays to identify ion selectivity and/or membrane disruption, and Hill plot analyses to quantify the total ion transport in our “catch all” KCl assay. The outputs of Path A are selectivity constants (*S*_anion_ or *S*_cation_), % lysis values from the SRB assay and EC_50_ values, defined as the concentration of transporter required to mediate 50% pH dissipation after 5 minutes. Transporters which do not mediate significant transport in our first assay (defined here as mediating <70% efflux) are evaluated as potential K^+^ uniporters by repeating our “catch all” KCl assay in the presence of CCCP (0.1 mol% w.r.t. lipid) to identify electrogenic transport. Transporters which exhibit an increase in observed activity (>70% efflux) are considered active, electrogenic transporters and progress *via* Path B for mechanistic evaluation and quantification. Transporters which do not meet this criterion follow Path C and are eliminated from the study as low potency or inactive compounds.

### Library 1 screening and evaluation

We applied the Workflow shown in Fig. 2 to identify transport activity within a library of 31 supramolecular, self-associating amphiphiles (SSAs), shown in Fig. 1c – designated as Library 1.

SSAs are small molecules or salts with amphiphilic properties and a number of distinct hydrogen bond donor and acceptor substituents.^50^ In aqueous environments, SSAs typically self-associate into structures including spherical aggregates and/or fibres.^51^ They have a range of well documented self-associative properties^51^ and a rich variety of biological function, including antimicrobial activity.^52–54^ Members from this class of compound have previously been shown to selectively recognise anionic lipids^55,56^ and also exhibit ion transport activity,^34,47^ hence we judged that a broader survey of ion transport activity within this library would be valuable and contribute to our understanding of the biological function of these molecules. The 31 compounds assayed here have been numbered as they are named within the Hiscock group library, to enable effective cross-referencing against the wider datasets held for these compounds. Most of these SSAs have been reported^34,50–52,56–61^ (although not for their ability to dissipate pH gradients), excluding 97 and 99 which are novel (with synthesis and characterisation provided in the SI, Section S1). SSAs 38 and 39 have previously been assessed for ion transport, with 38 reported to function as a low potency K^+^ uniporter in combination with Cl^−^ co-transport by Control compound AT in a chloride selective electrode (ISE) assay.^47^

**Fig. 3:**
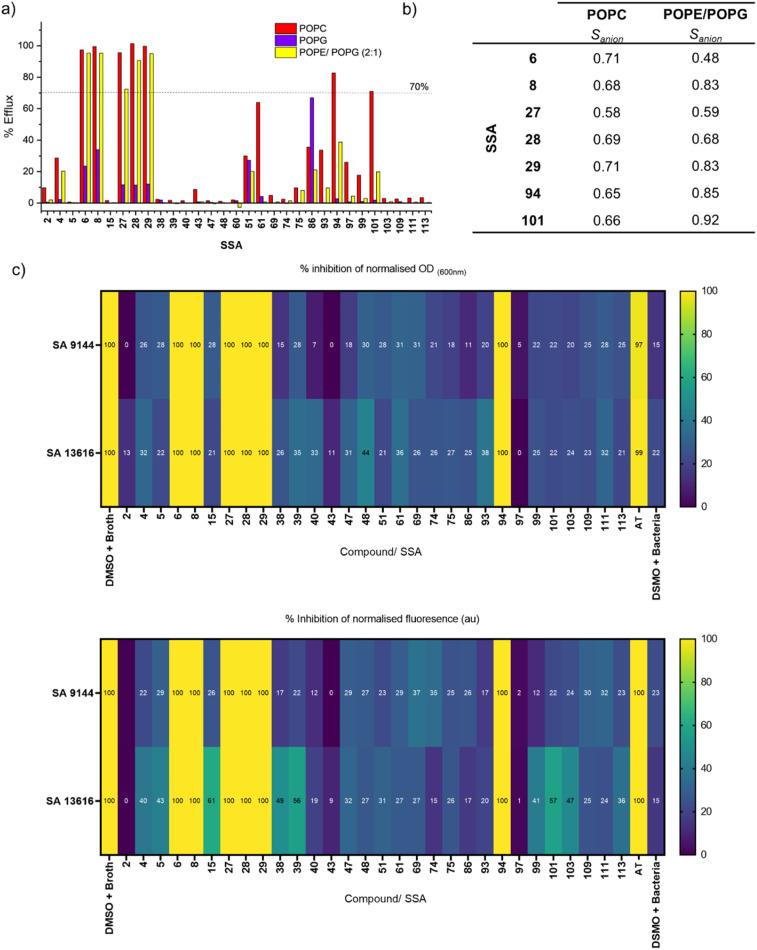
(a) Results from screening Library 1 (Fig. 1c) in the “catch all” KCl assay using POPC, POPG and POPE/POPG (2 : 1) vesicles, at 10 mol% with respect to lipid. The dotted line represents our cut-off criterion for further analysis (>70% efflux), used to identify the most active SSAs detailed in Fig. 3b; (b) *S*_anion_ values for the most active SSAs in Library 1 against POPC and POPE/POPG (2 : 1) tested at 10 mol% w.r.t. lipid; (c) heatmaps detailing the percentage of antimicrobial growth inhibition obtained for Library 1 and AT at 100 µM against *S. aureus* ATCC 9144 and *S. aureus* NCTC 13616 at 20 hours. Heatmaps display the percent inhibition of either optical density (turbidity or growth), and inhibition of fluorescence (metabolic activity) at 20 hours. All values were blank adjusted and normalised to the positive control for complete inhibition (DMSO + broth, to calibrate 100% inhibition of growth/metabolic activity).

According to the Workflow in Fig. 2, we initially screened the entire Library 1 in our “catch all” KCl assay, with the SSAs pre-dissolved in DMSO. We chose to perform this assay in vesicles composed of three different lipids; POPC (as a mimic of normal mammalian cell membranes), POPG (intended as a mimic of Gram positive bacteria, such as *S. aureus*),^62^ and a 2 : 1 mixture of POPE/POPG (intended to mimic the lipid extract of Gram negative bacteria such as *E. coli*).^63^ The results of this screening are shown in Fig. 3a. Many of the SSAs did not meet the criterion to progress *via* Path A (>70% efflux) in any lipid tested – this includes for example SSAs 2, 4 and 5. However, SSAs 6, 8, 27, 28, 29, 94 and 101 showed significant responses in POPC and, in some cases, POPE/POPG (2 : 1) and thus were progressed *via* Path A to further investigation as electroneutral transporters in these lipids. We also observed that the transport by all of these molecules was significantly reduced in POPG compared to the other two lipids trialled, demonstrating lipid selectivity. We hypothesise that the reduced activity in POPG may be due to interaction of these molecules with the POPG headgroup,^27,56^ which could slow transport, or due to a greater energetic barrier to transporting anions across membranes with a high proportion of anionic lipids. As a result, we did not further investigate their transport in POPG. Meanwhile, compounds such as 94 and 101 showed some selectivity for POPC over other lipids/combinations trialled. Unlike the other compounds in this library, SSA 86 showed higher transport efficiency in POPG compared to the other lipids tested, but did not meet the criterion for progression by Path A.

We were able to make some interesting observations based on the initial screening data in Fig. 3a. We found that SSA 6 was more active in our “catch all” assay than SSA 5, which could indicate that a longer chain length enabled more efficient pH dissipation. We have previously reported that the ethyl linker between the SSA anionic unit and the (thio)urea functionality resulted in a greater propensity for intramolecular hydrogen bond formation between these two substituents.^51^ This intramolecular bond formation in SSA 5 may serve to block the NH proton, which could inhibit transport; alternatively, the greater lipophilicity of this scaffold of the longer chain analogue may also play a role. SSAs 8 and 15 are similar in structure, and we unexpectedly found that 8 (the urea) was more active than 15 (the analogous thiourea) – this is contrary to previous observations that thioureas tend to be more effective transporters based on their greater lipophilicity,^64^ and could imply that the self-associative properties and resultant hydrogen bonded morphologies of these SSAs influence the transport efficiency. Finally, we also identified a single case for enantiomeric selectivity between SSAs 93 and 94, with 94 displaying markedly higher transport than it's enantiomer. We hypothesise that the importance of chirality in SSA transporter design may derived from the chirality of the phospholipids themselves, where ion transport is reliant on multiple complex formation events, including complexes formed with the phospholipid headgroups themselves, supporting recent results generated with Library 2.^34^ Paegel and co-workers have also observed that membranes composed of homochiral lipids are enantioselectivity permeable.^65^

Following our Workflow (Fig. 2), compounds that did not progress *via* Path A were re-evaluated as potential electrogenic transporters. We repeated our KCl screening experiment of all transporters at 10 mol% w.r.t. lipid in the presence of 0.1 mol% CCCP. An increase in observed transport would be interpreted as potential Cl^−^ or K^+^ uniport function (mimicking the response of Val, a K^+^ uniporter whose activity was enhanced by the addition of CCCP). However, we did not observe any appreciable enhancements in transport (Fig. S6).

As a result, SSAs 6, 8, 27, 28, 29, 94 and 101 progressed *via* Path A for further evaluation as electroneutral transporters in POPC and POPE/POPG, and the remaining SSAs were eliminated *via* Path C as low potency or inactive compounds.

It is notable that SSA 38, which we previously found to function as a low potency K^+^ transporter,^47^ did not register significant electrogenic transport here and thus was eliminated from our investigations at this stage. However, the assay used in our previous work (a modified chloride ion selective electrode assay) was significantly different from the present assay, employing higher salt concentrations and using both K^+^ and Cl^−^ gradients as driving forces for transport. Here, our HPTS assay only has a pH gradient to drive transport, with no K^+^ or Cl^−^ gradient. While this driving force is sufficient to reveal highly potent cation transporters such as Val (Table 1), it may mean that low potency transporters such as 38 fall below the threshold. We therefore suggest that our new assay is well-suited for identifying high potency ion transporters, and could impose a useful cut-off when searching for therapeutically promising molecules. Identifying lower potency transporters and investigating their mechanisms may require the use of other assays that incorporate defined ion gradients, and/or higher ionic strength conditions.

Moving forwards in our Workflow, we next subjected our active compounds (that met the threshold of >70% efflux) to mechanism and quantification studies detailed in Path A. Ion selectivity experiments in POPC and POPE/POPG (2 : 1) yielded the data and *S*_anion_ values shown in Fig. 3b and Tables S2, S5. The majority of compounds tested showed anion selectivity (*S*_anion_ > 0.5) in both lipids, with the exception of 6 which displayed no selectivity in POPE/POPG (2 : 1). In most cases, the selectivity was lower than our benchmarking anion transport systems (0.1 mol% Prod and 0.6 mol% AT in POPC). This low anion selectivity highlights that the observed pH dissipation is not completely dependent on the presence of chloride. Our SRB assays (Fig. S7) revealed that the majority of compounds in this series mediate relatively low levels of lysis (<20%) under these conditions, hence we believe that only a minor fraction of the observed pH dissipation can be attributed to lysis. One exception was SSA 8, which apparently mediated approximately 60% lysis in POPE/POPG (2 : 1) vesicles, but only around 10% lysis in POPC, showing an interesting lipid-dependency on the mode of action. These experiments suggest that within this class of compound, distinct ion-transport and lysis mechanisms may occur simultaneously, but vary in their contribution to the overall pH dissipation.

We also sought to quantify the ion transport in our “catch all” KCl assay by SSAs 6, 8, 27, 28, 29, 94 and 101 which had progressed *via* Path A by performing Hill analyses to produce EC_50_ values. To identify the optimum concentration range for each system, we first performed trial dose–response experiments over a large concentration range for each SSA in POPC, POPG and POPE/POPG (2 : 1). The data is shown in Fig. S8. Correlating with the screening experiments, we saw limited transport across this series in POPG, and hence we did not pursue further quantification in this lipid due to low potency. We also observed lower than expected transport by SSAs 27, 94 and 101 in POPC and POPE/POPG (2 : 1) compared to our screening experiment (here, <50% efflux at 10 mol%). This could reveal that the concentration of the stock DMSO solutions (which are varied within the dose–response experiment) affects the observed transport, which could indicate aggregation into less active species at higher concentrations. As such, we did not pursue further quantification of transport in these systems due to the low potency.

Following this initial concentration screen, we performed triplicate Hill analyses for SSAs 6, 8, 28 and 29 in POPC and POPE/POPG (2 : 1) (Fig. S10). We also included our Control – AT, in this panel to act as an activity benchmark, validating the results of the individual assays. The results, shown in Table 2, indicate that these transporters (6, 8, 28, 29) are all similarly active in these two vesicle compositions, and that AT is approximately an order of magnitude more potent than the SSAs investigated.

**Table 2:** Results from the triplicate Hill plot analysis of AT, 6, 8, 28 and 29 in both POPC and POPE/POPG (2 : 1) vesicles. Errors represent the standard deviation

Control/SSA	EC_50_ (POPC)/mol% w.r.t. lipid	EC_50_ (POPE/POPG)/mol% w.r.t. lipid
6	3.5 ± 0.2	3.5 ± 0.2
8	1.7 ± 0.3	2.1 ± 0.2
28	3.7 ± 0.2	5.3 ± 0.3
29	4.3 ± 0.8	5.8 ± 0.4
AT	0.225 ± 0.008	0.185 ± 0.009

In summary, our increased throughput Workflow identified seven active transporters within Library 1. The highest transport efficiencies were observed in POPC and POPE/POPG (2 : 1), with lower transport observed in POPG. Most active compounds displayed some degree of anion selectivity and relatively low levels of lysis/membrane rupture.

### Antimicrobial testing

In parallel to investigating the properties of Library 1 *via* our vesicle Workflow, we also investigated the antimicrobial activity of the same compounds with the aim of identifying correlations between the outcomes of these experiments. We hypothesised that the dissipation of proton gradients across the inner membrane of bacteria may cause short-term changes in metabolism and energy homeostasis. In many bacterial species – including those used within the scope of these studies, NADH is consumed to drive the translocation of protons across the inner membrane and the subsequent proton-motive force drives ATP synthesis. Proton transport provides a mechanism where NADH is consumed without generating ATP.^66^ The short-term depletion of NADH (after 5 hours of incubation with potential therapeutics), indicating a reduction in metabolic activity, can be observed using a resazurin fluorescence assay (also known as AlamarBlue®), in which a non-fluorescent indicator is reduced by NADH to become fluorescent. This assay has been used to investigate antimicrobial efficacy in Gram-positive bacteria,^67^ Gram-negative bacteria,^67,68^ fungal^69^ and eukaryotic cells.^70^ Long term toxicity (after 20 hours incubation with SSAs) was also investigated using the resazurin fluorescence assay as well as complimentary optical density (OD) measurements obtained at 600 nm, which is an established metric of bacterial growth.

We initially screened all SSAs within Library 1 (Fig. 1c) plus AT at a concentration of 100 µM against two Gram-positive *S. aureus* strains, ATTC 9144 and NCTC 13616. Gram-positive bacteria are not protected by an outer membrane, and hence we reasoned that they were the most accessible target for potential membrane-active antimicrobials. We selected one strain that is susceptible to clinically relevant antibiotics (ATCC 9144) and another strain that is multidrug resistant including methicillin (NCTC 13616). Methicillin resistant *S. aureus* (MRSA) are listed in the WHO bacterial priority pathogens 2024.^71^ To mirror the conditions used in our transport experiments, the transporters were delivered as DMSO solutions to bacterial cells to final assay concentration of 10% DMSO, a concentration of DMSO which does not inhibit bacterial growth on its own.

Typically, antimicrobial susceptibility testing measures the turbidity, or the growth of bacteria, of a solution at OD 600 nm over a period of 20 hours. The growth of bacteria in the presence of a compound is compared to the growth in the media alone. A reduction of ≥90% in growth in the presence of a compound is deemed inhibition or compound activity. The results from these studies are presented in Fig. 2c, and show that remarkably, the six most active SSAs identified by our vesicle Workflow inhibited microbial growth by ≥90% compared to the growth of cells in the absence of SSA, while all other compounds in this library were inactive or had much lower potency.

Having identified six antimicrobial SSAs (6, 8, 27, 28, 29 and 94), we then performed experiments to determine minimum inhibitory concentration (MIC) values for all of the compounds identified in this screen plus one randomly selected inactive SSA (47) against 2 different bacterial species (3 different strains); *S. aureus* (ATCC 9144 and NCTC 13616) and *E. coli* (NCTC 12923). These strains were chosen to include two Gram-positive strains (the *S. aureus* strains), which have a single phospholipid bilayer membrane, compared to the *E. coli* as a Gram-negative strain with a periplasmic space between an inner and outer membrane. The inner membrane in Gram-negative bacteria is less accessible to the SSAs (and many other antimicrobial compounds) due the presence of an outer cell membrane. We specifically chose *E. coli* as our Gram-negative species due to the transport activity of our compounds in the lipid mixture designed to mimic the *E. coli* inner membrane (POPE/POPG (2 : 1)). SSAs were delivered as DMSO solutions, which were directly added to prepared inocula, in a modification of standard MIC protocols that we term an inverted MIC protocol. The results are summarised in Table 3, and supporting data is available in the SI Section S4.

**Table 3:** MIC values (µM) for active SSAs (6, 8, 27, 28, 29, 94 plus AT), plus one randomly selected inactive compound (47)

Compound/SSA	*S. aureus*		*E. coli*
	ATCC 9144	NCTC 13616	NCTC 12923
6	125.0	31.25	>1000
8	31.25	7.813	>1000
27	62.50	31.25	>1000
28	125.0	31.25	>1000
29	31.25	62.50	>1000
47	>1000	>1000	>1000
94	250.0	125.0	>1000
AT	31.25	31.25	>1000

A range of antimicrobial activity was observed against the two *S. aureus* strains tested, with a greater activity observed for SSAs against the clinically relevant MRSA strain NCTC 13616, over the drug susceptible strain ATCC 9144. The SSA demonstrating the greatest antimicrobial activity against *S. aureus* NCTC 13616 was SSA 8 with an MIC value of 7.8 µM. This SSA was also identified as the most potent transporter from Library 1 in POPC and POPE/POPG (2 : 1) vesicles; however, there is no quantitative correlation observed between the antimicrobial MIC (Table 3) and EC_50_ (Table 2) values obtained. We also note that AT was a significantly more potent anion transporter than SSA 8 in our vesicle models, yet with an MIC of 31.25 µM demonstrated a much lower antimicrobial activity against *S. aureus* NCTC 13616. This demonstrates that links between ion transport efficacy and biological activity should be very carefully considered.

None of the compounds tested showed appreciable activity against the Gram-negative *E. coli* (NCTC 12923) strain. This reduced susceptibility is consistent with the well-known intrinsic resistance of Gram-negative bacteria relative to Gram-positive organisms, which is frequently attributed to the permeability barrier provided by the outer membrane.^72^ This protection can be the result of poor penetration of drug molecules through the outer membrane and/or the presence of efflux pumps which can reamove drug molecules before they can fulfil their intended function. Most antibiotics permeate the outer membrane *via* porin channels and pores, whose polar interior favours the permeation of polar drug molecules.^73^ Hydrophobic antibiotics can penetrate through the outer membrane layer, but the presence of lipopolysaccharides in the outer leaflet impose a substantial barrier to this route.^72^ Taken together, these considerations suggest that the lack of observed activity of the active ion transporters in Library 1 against Gram-negative bacteria may reflect limited outer membrane penetration, inefficient uptake *via* porins and/or rapid excretion *via* efflux pumps.

We also carried out conventional MIC assays, diluting the compounds in TSB before addition to the prepared inocula. We found no difference in modal MIC values from our inverted protocols, except for SSA 8, which exhibited a 16-fold decrease in antimicrobial activity against *S. aureus* ATCC 9144, and a 64-fold decrease in antimicrobial activity against *S. aureus* NCTC 13616 under these traditional assay conditions. This suggests that the antimicrobial efficacy of this SSA significantly changes when it is not in a DMSO or lipid rich environment. We know from our previous work that in DMSO this SSA exists predominantly as single or dimeric species, which would aggregate upon addition to an aqueous environment.^50,51,58^ These data suggest that our inverted protocol could find utility in assessing the antimicrobial activity of highly lipophilic synthetic ion transporters, which may be poorly compatible with aqueous buffers. However, we note that DMSO has been reported to increase membrane permeability,^74^ and hence despite its widespread use in ion transporter testing we should not assume that it’s influence on the outcomes of our experiments is negligible, and DMSO concentrations must be kept low during biological testing. For future efforts to reduce our reliance on DMSO, the delivery of hydrophobic antibiotics such as tetracycline has been achieved using a range of nanocarriers,^75^ and similar approaches may prove useful for the formulation of synthetic ion transporters.^76^

To further probe the potential aggregation of our active SSAs under the conditions of our antimicrobial assays, we carried out dynamic light scattering (DLS) experiments with all active transporters at 0.1 mM in TSB/5.0% DMSO (see SI, Section S5) and compared the results to those obtained with the TSB/5.0% DMSO medium alone. The results were inconclusive, as control experiments showed that large species present in the TSB buffer itself dominated the scattering signal, obscuring any aggregates formed by the SSAs under these conditions. However, we note that SSA 8 has previously been reported to form relatively unstable aggregate species in 1 : 19 EtOH : H_2_O solution at a concentration of 5.56 mM, with a significantly higher critical micelle concentration than analogues in which the pyridinium counterion is substituted with tetra-*n*-butylammonium.^51^ We therefore suggest that this unusual aggregation behaviour (compared to other, comparable SSAs) may impact the organisation of the SSA as it arrives at the membrane, and it's subsequent membrane incorporation.

Finally, since our active transporters mostly displayed some anion selectivity (Fig. 2b) in our vesicle Workflow, we aimed to investigate the anion selectivity of the observed antimicrobial function. We performed cell viability experiments using resazurin staining in both a chloride-rich buffer HBSS (Hanks buffered salt solution), and an analogous chloride-free buffer in which all chloride anions were replaced with gluconate. *S. aureus* NCTC 13616 was selected for this work and we validated that while this organism does not grow in either buffer, it is also not killed in these buffers over 20 hours of incubation. We further validated that the resazurin stain could provide adequate changes in fluorescence intensity (FI) within 2 hours. The full protocol and data are included in SI, Section S4.2. However, we did not observe any significant impact on the observed antimicrobial action of our molecules (including AT) by removing the chloride. Therefore, we cannot attribute the observed growth and metabolism inhibition to selective anion transport for any of the compounds tested here, which could instead imply that a combination of transport or lysis pathways contribute to the overall antimicrobial function.

In summary, we found that the active transporters from Library 1 (as identified by our vesicle Workflow) showed good antimicrobial activity against two strains of *S. aureus*. This demonstrates the applicability of our vesicle Workflow to qualitatively identify compounds with a high chance of exhibiting antimicrobial activity.

However, the results of ion transport experiments in different lipids did not map onto the outcomes of our antimicrobial experiments as expected. Transport in our model *E. coli* vesicles (POPE/POPG 2 : 1) was high, yet antimicrobial activity against *E. coli* was low. We rationalised the low activity against *E. coli* by considering that penetration through the outer membrane and cell wall of the Gram-negative bacteria could be a limiting factor. However, transport in our model *S. aureus* vesicles (POPG) was low yet the antimicrobial activity against the *S. aureus* strains was high. We consequently sought to evaluate the specific lipid compositions of the two *S. aureus* strains to seek better understanding of how the membranes of our model systems and the real bacterial cells may differ.

### Phospholipid composition of bacterial cells

**Fig. 4:**
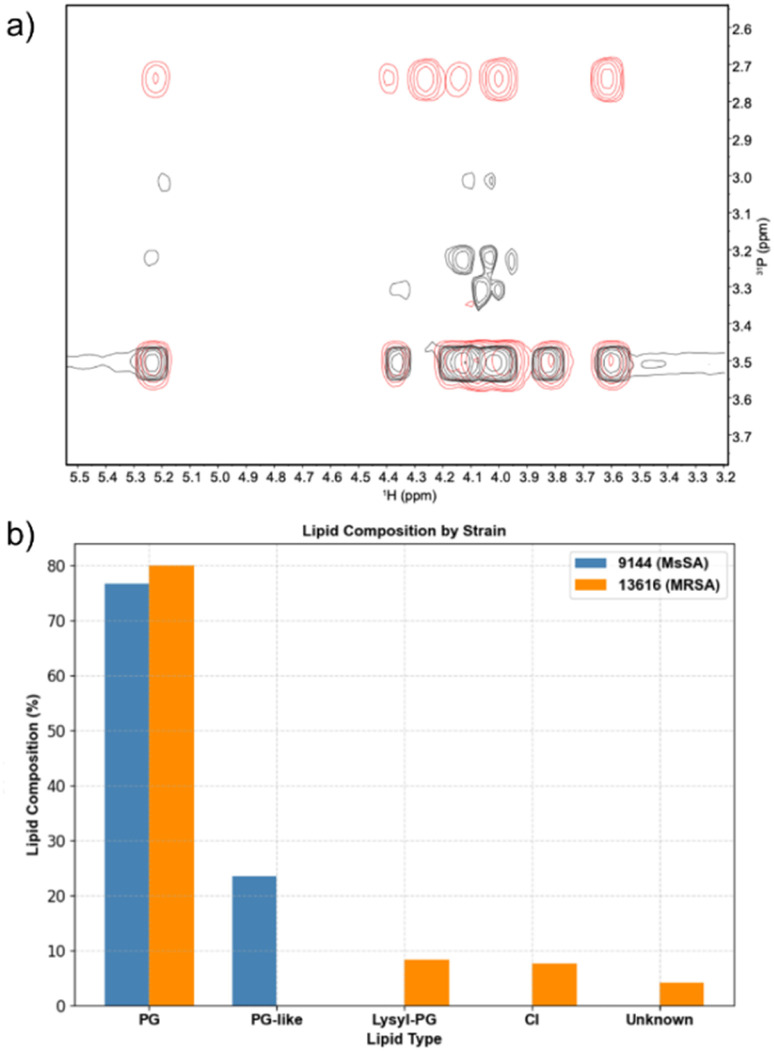
(a) Overlay of ^31^P HSQC spectra of phospholipids extracted from *S. aureus* ATCC 9144 (red) and *S. aureus* NCTC 13616 (black); (b) differences in total phospholipid composition between *S. aureus* ATCC 9144 (blue) and *S. aureus* NCTC 13616 (orange).

Phospholipids were extracted from cell cultures of *S. aureus* ATCC 9144 and *S. aureus* NCTC 13616 using the Folch method (see SI Section S6) and analysed using ^31^P NMR spectroscopy analysis.^77^ The results are shown in Fig. 4. We found that *S. aureus* ATCC 9144 exhibited a total phospholipid membrane composition consisting of 77% PG and 23% of a PG-like lipid, with cardiolipin present only in trace amounts below the limit of detection. In contrast, *S. aureus* NCTC 13616 contained a comparable proportion of PG, accompanied by 8% lysyl-PG, 8% cardiolipin, and 4% unidentified phospholipid.

Given the low observed activity of Library 1 in POPG vesicles, we conclude that simply matching the phospholipid headgroup of our vesicles to the major component of the membranes of the bacteria of interest does not produce the best indication of antimicrobial activity, and instead our results in POPC and POPE/POPG (2 : 1) vesicles yielded a better qualitative indication of antimicrobial activity. This may be a consequence of the simplicity of our model membranes, which lack features of bacterial membranes such as lipoproteins and glycolipids, which can significantly influence the organisation and structure of the membranes.^78^ This finding may also demonstrate that other potential modes of action – such as lipid headgroup binding – may contribute to the observed antimicrobial activity.^34^

### Library 2 evaluation

Having established the utility of our vesicle Workflow in identifying potential antimicrobial candidates from Library 1, we next sought to use our assays to assess the function of a set of SSAs with established biological activity. There are numerous reported links between ion transport and antimicrobial/anticancer functions (as discussed in the introduction), and hence we aimed to evaluate a subset of molecules with both established antimicrobial and anticancer activity *via* our Workflow. On this basis, we identified Library 2 as a promising subset of molecules for analysis. A recent study has reported a range of anticancer and antimicrobial data for the SSAs within Library 2 (Fig. 1c), alongside preliminary experiments showing ion transport (antiport) and membrane disruption events in synthetic membranes.^34^ Specifically, SSAs 30, 56, 57, 72, 73 and racemic mixtures of 56/57 and 72/73 have previously been found to exhibit both antimicrobial and anticancer efficacy in experiments against multiple drug susceptible and resistant *S. aureus* and ovarian cancer cells, with selectivity over normal human cells.^34^ Our aim here was to elucidate any potential contributions that ion transport or lysis processes may play towards the established cytotoxicity profiles of these molecules. Our aim here was to elucidate any potential contributions that biologically relevant ion transport or lysis processes may play towards the established cytotoxicity profiles of these molecules, and to elucidate any influence that the lipid composition of the vesicle bilayers and the concentration of the molecules themselves played on the observed function(s).

The earlier biological experiments for Library 2 had been performed using water/EtOH (95 : 5) as a delivery solvent rather than DMSO, we also performed most of our ion transport experiments using this solvent medium. However, since preliminary ion transport and patch clamp experiments (both in PC bilayers) had been performed using DMSO as the delivery solvent, we performed an additional set of Hill plot experiments in POPC using DMSO for comparison. To maximise both the cost and time efficiency of these experiments, we took the following steps: (i) our positive controls in these experiments were either AT (ion transport) or Triton (lysis and 100% pH dissipation) as appropriate, which are less costly than Prod and Val; (ii) we designed a standardised plate layout for screening and Hill plot experiments; (iii) we wrote a script to automate and hence streamline the raw data processing based on our standardised plate layouts (details can be found in the SI Section S7). This script enabled the rapid processing of raw data files produced by the platereader (in .csv format) into processed and fitted Hill plot data. We have made our script available for download *via* Github.^79^

**Fig. 5:**
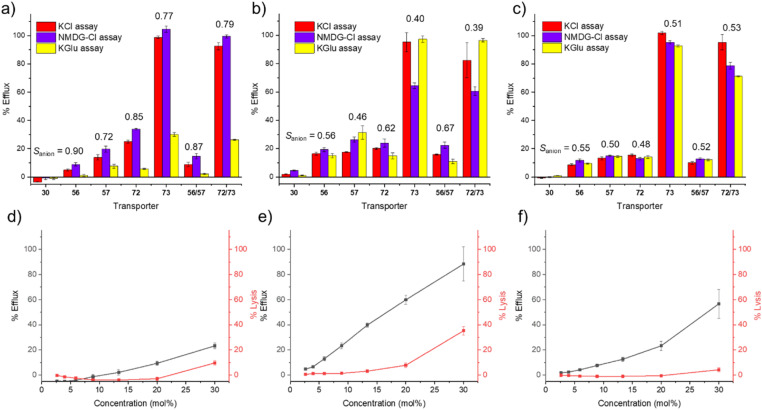
(a)–(c) Results from screening Library 2 at 10 mol% in the KCl (catch-all), NMDG–Cl and KGlu assays; (a) screening results in POPC vesicles; (b) screening results in POPG vesicles; (c) screening results in POPE/POPG (2 : 1) vesicles. *S*_anion_ values for each transporter in each lipid are shown above the data, excluding 30 which was inactive at this concentration in all buffers trialled. (d)–(f) Results from dose–response KCl and SRB assays for compound 57; (d) dose–response experiments in POPC vesicles; (e) dose–response experiments in POPG vesicles; (f) dose–response experiments in POPE/POPG (2 : 1) vesicles. Each data point represents a minimum of three repeat measurements, and error bars represent standard deviations.

In line with our standardised Workflow (Fig. 2), we initially screened the activity of SSAs 30, 56, 57, 72, 73 and racemic mixtures of 56/57 and 72/73 at 10 mol% in our catch-all KCl assay in POPC, POPG and POPE/POPG (2 : 1). The results of this screening are summarised in Fig. 5a–c. We found that 30 was essentially inactive at this concentration under all of these experimental conditions; however, the other SSAs in this series showed a range of responses. Given the established biological activity of these molecules, we chose to remove the criterion of achieving >70% pH efflux and progressed SSAs 56, 57, 72, 73 and the racemic mixtures through Path A to our mechanism and quantification studies.

It is noteworthy that SSA 30 (Library 1) displayed such low activity here, whereas SSA 29 showed good activity within our Library 1 experiments (both structures are shown in Fig. 1). However, previous work has demonstrated that both protonation state and the presence/nature of the counterion can influence the aggregation state of SSAs, and we speculate that this may have caused this apparent difference in activity. To a lesser extent, we also observed this effect with SSAs 101 and 103 in our initial Library 1 screening (Fig. 3a).

Moving forwards with our Workflow, we then performed ion selectivity experiments in our three lipid mixtures using SSAs 56, 57, 72, 73 and the racemic mixtures at 10 mol%, and the results of these assays, and *S*_anion_ values, are shown in Fig. 5a–c. In POPC vesicles, we uniformly observed a reduction in transport efficiency in chloride-free buffer whilst the counter cation had little effect on transport (Fig. 5a). This is illustrated by the *S*_anion_ values, which indicated that these transporters had good anion selectivity at this concentration in POPC. However, this trend was not replicated in our other lipid mixtures. In POPG and POPE/POPG (2 : 1) vesicles, there was typically little variation across the three assays, which could imply that non-selective ion transport or lysis pathways are in play. This finding highlights the importance of considering influence of the lipid composition on the function of potential ion transporters, as molecules that exhibit selective transport in one type of lipid may perform different mechanisms in others.

SRB experiments at this concentration did not reveal significant lysis or membrane disruption (see Fig. 5e–g and SI Fig. S16, S17), but we note that patch clamp experiments had revealed evidence of multiple, distinct ion transport pathways,^34^ including potential “mass transport” events – which would yield less selective transport.

Moving through our Workflow (Fig. 2), we attempted to quantify the overall pH dissipation efficiency of 56, 57, 72, 73 and racemic mixtures of 56/57 and 72/73*via* Hill plot experiments in all three lipid mixtures. However, many of the dose–response profiles we obtained were not in line with a typical Hill analysis. We typically expect to see an approximately sigmoidal curve between 0% to 100% efflux – good examples of this are the responses of 72, 73 and 72/73 in POPC (Fig. S12–S14). However, in some cases – particularly in POPG and POPE/POPG (2 : 1) vesicles – we saw a sharp increase in transport above a particular concentration threshold. An example is given in Fig. 5f for SSA 57 in POPE/POPG (2 : 1). We speculated that this sudden increase in transport at the highest concentrations of SSA could be due to the onset of another pathway, such as lysis or mass transport, and hence we took the additional step of performing dose–response SRB leakage assays, in which the concentration of transporter was varied in all three lipid mixtures. We then compared the concentration dependent transport with the concentration lysis profiles; illustrative examples for SSA 57 are shown in Fig. 5d–f, and comparable data for all SSAs in this series are provided in Fig. S16 and S17. We typically observed low levels of SRB leakage in POPC vesicles for all of the molecules in this series, which supports the hypothesis that these molecules can mediate selective ion transport in PC bilayers. In contrast, we observed higher levels of lysis in POPG vesicles, but often with a different concentration dependence to the observed overall pH dissipation. This suggests that these molecules may be able to mediate both ion transport and lysis at different concentrations. Finally, we generally observed lower levels of lysis in POPE/POPG (2 : 1) vesicles, excluding SSA 73 and the racemic mixture of 72/73. However, considering the apparent lack of selectivity observed in the screening experiments, and the unusual shape of the KCl dose–response curves, we suggest that a combination of different transport mechanisms (such as mass transport events) may be responsible for the response in the KCl assay in POPE/POPG (2 : 1).

From these results, we conclude that the overall pH dissipation mediated by these SSAs is highly lipid specific, but also that the mechanisms by which these compounds function is also lipid specific. We suggest that our observed “transport” is in fact the net result of a combination of different transport and lysis events, which may all have their own distinct concentration dependence profiles. This highlights the importance of carefully considering both the influence of different lipids and different potential mechanisms of action within our Workflow.

To quantify the apparent transport by these SSAs, we attempted to fit our KCl dose–response data to the Hill equation to derive EC_50_ values using both our manual protocol (using a combination of Microsoft Excel and Origin 2019) and our automated Python script. An overview of the development of our script and a comparison of the manual and automated data processing protocols and outputs can be found in the SI, Section S7. However, given the unusual profiles of the KCl dose–response data in POPG and POPG/POPE (2 : 1) vesicles, which we had hypothesised may be due to a combination of different transport and lysis mechanisms, in many cases the fit to the Hill equation was relatively poor due to spikes in activity at high concentration. As such, we present here a comparison of the EC_50_ values in POPC (other results and attempted fittings are presented in the Fig. S12 and S13). We also repeated our KCl Hill plot experiments in POPC using DMSO as a carrier solvent, since previously reported Cl^−^/NO_3_^−^ antiport and patch clamp experiments had been performed from DMSO solutions. The results are shown in Table 4.

**Table 4:** A summary of the EC_50_ values (manual fitting) determined for active transporters in the KCl assay in POPC vesicles, using both water/EtOH (95 : 5) and DMSO as the delivery solvent. Errors derived from the fitting in Origin 2019 < 20%

Control/SSA	EC_50_ (water/EtOH 95 : 5)/mol% w.r.t. lipid	EC_50_ (DMSO)/mol% w.r.t. lipid
56	n.d.	n.d.
57	n.d.	n.d.
56/57	n.d.	n.d.
72	32.9	29.9
73	4.4	2.9
72/73	7.8	4.8

The data shown in Table 4 suggests that 73 is by far the most potent transporter in this series in POPC vesicles and is approximately an order of magnitude more active than its enantiomer (72). This trend is replicated in both delivery solvents trialled, indicating that the observed transport is not strongly influenced by the choice of delivery solvent in this case and that transport efficiency can be maintained in delivery solvents with a high (95%) water content.

Encouragingly, our automated script produced similar outputs to our manual fitting, both in terms of the calculated constants (EC_50_) and in terms of the fit to the experimental data (see SI, Section S7). Given the close agreement in outputs, our automated data processing script can offer a number of advantages over our manual protocol: (1) the analysis is much faster, particularly for handling Hill plot data; (2) the raw data file produced by the platereader (in .csv format) can be fed directly into the script, along with key variables such as transporter identities and concentrations, to produce processed, plotted and fitted data, including the key outputs of the fitting (EC_50_ values). Compared to fitting to standard equations in Origin, our process could also enable greater control/flexibility over the fitting, and could enable re-fitting and computational analysis of the quality of fit across large libraries. This could be helpful in identifying outliers with non-typical Hill behaviour, such as the examples noted in this work which we attribute to a potential interplay of different transport and lysis mechanisms. Finally, this process produces a computationally accessible bank of data that could support more in depth structure–activity relationship modelling in the future.

### Correlation with antiport data and biological activity

Finally, we sought to compare the outcomes of our testing with the previously established biological activity of these molecules.^34^ Testing against ovarian cancer cells had established that 73 and the racemic mixture of 72/73 exhibited the greatest activity against both cisplatin susceptible and resistant cancer cell lines. This correlates with our finding that these transporters/mixtures displayed the greatest efficacies in both our ion transport/lysis experiments. However, antimicrobial testing against the methicillin susceptible *S. aureus* ATCC 9144 strain indicated that 72 was more active than 73, which does not correlate with the outcomes of our experiments. In other strains, these two enantiomers displayed similar activities. We therefore suggest that while ion transport and/or lysis processes could contribute to the observed biological activity, they may not be the sole determinant. This correlates with our findings from our “catch all” screening of Library 1, where we found that the outcomes of our vesicle assays were a good qualitative indicator of biological function, but no quantitative correlation was observed.

## Conclusions

In conclusion, we have developed an increased throughput Workflow (Fig. 2) to identify biologically relevant pH dissipation and membrane lysis functions across libraries of novel compounds in synthetic vesicle membranes. Our Workflow contains distinct steps to: (1) rapidly screen novel compound libraries in a “catch all” assay (an adaptation of the literature HPTS assay); (2) identify key mechanistic features, such as ion selectivity and membrane rupture; (3) quantify the pH dissipation *via* Hill plot analysis. We have also developed an automated data processing pathway to streamline our data processing and build repositories of computationally accessible ion transport data. The improved throughput of our methods compared to conventional approaches provides a platform for investigating the influence of experimental parameters such as the lipid composition of the vesicles, and here we have systematically evaluated the influence of the phospholipid headgroups within the vesicle on the potency and mechanism of the transporters identified. We have compared the outputs of our experiments to key biological experiments to improve our understanding of how ion transport in synthetic vesicles maps onto behaviour and cytotoxicity at a cellular level.

Our experiments with the 31 compounds in Library 1 identified seven previously undiscovered, electroneutral transporters which showed promising ion transport in POPC and POPE/POPG (2 : 1) vesicles and reduced activity in POPG vesicles. Mechanistic investigations indicated that most transporters in this series displayed a degree of anion selectivity, and low levels of lysis or membrane rupture were detected. The entire library was subjected to antimicrobial screening, with six of these SSAs identified as electroneutral transporters also found to exhibit antimicrobial activity against two strains of *S. aureus*. This demonstrates the promise of our Workflow in identifying potential leads for antimicrobial investigation. The most potent SSA transporter was SSA 8, and this SSA also displayed the most significant antimicrobial activity within Library 1.

Our experiments with the five SSAs in Library 2 enabled us to identify and characterise ion transport processes mediated by these compounds in different lipids, and to begin to understand the influence of the choice of lipid on the complex interplay of potential mechanisms that these molecules can facilitate. While we observed anion selective transport in POPC, we found that the transport selectivity was significantly reduced in POPG and POPE/POPG (2 : 1). By performing dose-dependent transport and lysis assays, we saw evidence that several different transport and/or lysis profiles may operate, each with their own distinct concentration profiles. This correlates with data from patch clamp experiments, which showed that this class of compounds can mediate a number of distinct types of transport event, including “mass transport”. The most potent transport identified was compound 73, and this correlates with the finding that this compound had the highest cytotoxicity against ovarian cancer cells.^34^

Our studies also revealed some additional findings. Firstly, the active transporters in Library 1 showed low ion transport in POPG, which we expected to be the best model of *S. aureus* bacterial cells, yet showed strong antimicrobial activity against two *S. aureus* strains. This finding highlights that matching the phospholipid headgroup of the vesicles to the major components of the bacterial cell membrane does not necessarily provide the best model. Additionally, while we observed anion selective transport in our vesicle experiments, we could not observe significant anion-dependence on the antimicrobial function. Meanwhile, in our vesicle study of Library 2, we observed that SSA 73 (derived from d-phenylalanine) was more active than it's enantiomer, SSA 72. However, antimicrobial testing against the methicillin susceptible *S. aureus* ATCC 9144 strain indicated the opposite trend, while in other strains the two enantiomers performed similarly.^34^

We therefore acknowledge that, while our Workflow shows promise in qualitatively identifying novel ion transporters as therapeutic leads, further work is required to enable a quantitative prediction of potency and mechanism in the target cells of interest. There are undoubtedly more questions to be asked. By standardising and increasing the throughput our Workflows, it will become possible to probe a much broader range of hypotheses, enhancing our understanding of these systems, and ultimately, accelerating their therapeutic translation.

## Author contributions

KY: conceptualisation, data curation, formal analysis, investigation, methodology, resources, validation, visualisation, writing – original draft, writing – review & editing; CM: data curation, formal analysis, investigation, methodology, visualisation, writing – original draft, writing – review & editing; LJW: investigation, resources, visualisation; FWM: investigation, data curation, formal analysis, writing – review & editing; TLA: investigation, data curation, formal analysis, software, writing – original draft writing – review & editing; PIAP: investigation, resources; OBK: investigation, resources; MR: investigation, data curation; KLFH: investigation, resources; HAK: investigation, resources; JMS: funding acquisition, supervision, writing – review & editing; JLOR: funding acquisition, supervision, visualisation, writing – original draft, writing – review & editing; CKH: conceptualisation, data curation, formal analysis, funding acquisition, investigation, methodology, project administration, supervision, visualisation, writing – review & editing; JRH: conceptualisation, funding acquisition, investigation, methodology, project administration, resources, supervision, visualisation, writing – review & editing; CJEH: conceptualisation, data curation, formal analysis, funding acquisition, investigation, methodology, project administration, supervision, visualisation, writing – original draft, writing – review & editing.

## Conflicts of interest

There are no conflicts to declare.

## Supplementary Material

SC-OLF-D5SC09781A-s001

## Data Availability

The supporting data has been provided as part of the supplementary information (SI). The code for automated data processing can be found at https://github.com/ta1u18/EC50-high-throughput-workflow. Supplementary information: details of synthesis and characterisation, protocols and results of ion transport experiments, antimicrobial protocols and data, dynamic light scattering data and details of the automated data processing. See DOI: https://doi.org/10.1039/d5sc09781a.

## References

[cit1] Feo E., Gale P. A. (2024). Curr. Opin. Chem. Biol..

[cit2] Rathnaweera U. M. C., Chowdhury S. M., Salam R., Busschaert N. (2025). Chem. Rev..

[cit3] Li H., Valkenier H., Thorne A. G., Dias C. M., Cooper J. A., Kieffer M., Busschaert N., Gale P. A., Sheppard D. N., Davis A. P. (2019). Chem. Sci..

[cit4] Huang W., Jia C., Ren C. (2025). ChemMedChem.

[cit5] Mondal A., Barik G. K., Sarkar S., Mondal D., Ahmad M., Vijayakanth T., Mondal J., Santra M. K., Talukdar P. (2023). Chem.–Eur. J..

[cit6] Gianotti A., Capurro B., Delpiano D., Mielczarek M., García-Valverde M., Carreira-Barral I., Ludovico A., Fiore M., Baroni M., Moran O. (2020). et al.. Int. J. Mol. Sci..

[cit7] Brennan L. E., Luo X., Mohammed F. A., Kavanagh K., Elmes R. B. P. (2025). Chem. Sci..

[cit8] Shibamura-Fujiogi M., Wang X., Maisat W., Koutsogiannaki S., Li Y., Chen Y., Lee J. C., Yuki K. (2022). Commun. Biol..

[cit9] Maslowska-Jarzyna K., Ojah E. O., Korczak M. L., Chmielewski M. J., Busschaert N. (2025). ACS Omega.

[cit10] Maslowska-Jarzyna K., Cataldo A., Marszalik A., Ignatikova I., Butler S. J., Stachowiak R., Chmielewski M. J., Valkenier H. (2022). Org. Biomol. Chem..

[cit11] Share A. I., Patel K., Nativi C., Cho E. J., Francesconi O., Busschaert N., Gale P. A., Roelens S., Sessler J. L. (2016). Chem. Commun..

[cit12] Das S., Karn R., Kumar M., Srimayee S., Manna D. (2024). Org. Biomol. Chem..

[cit13] Dey S., Patel A., Raina K., Pradhan N., Biswas O., Thummer R. P., Manna D. (2020). Chem. Commun..

[cit14] Carreira-Barral I., Rumbo C., Mielczarek M., Alonso-Carrillo D., Herran E., Pastor M., Del Pozo A., García-Valverde M., Quesada R. (2019). Chem. Commun..

[cit15] La Cognata S., Armentano D., Marchesi N., Grisoli P., Pascale A., Kieffer M., Taglietti A., Davis A. P., Amendola V. (2022). Chemistry.

[cit16] Mondal A., Siwach M., Ahmad M., Radhakrishnan S. K., Talukdar P. (2024). ACS Infect. Dis..

[cit17] Akhtar N., Conthagamage U. N. K., Bucher S. P., Abdulsalam Z. A., Davis M. L., Beavers W. N., García-López V. (2024). Mater. Adv..

[cit18] Umashankar B., Pazderka C., York E., King A., Rahman M. K., Choucair H., Bourget K., Gale P. A., Rawling T., Murray M. (2025). J. Pharm. Sci..

[cit19] Tapia L., Pérez Y., Carreira-Barral I., Bujons J., Bolte M., Bedia C., Solà J., Quesada R., Alfonso I. (2024). Cell Rep. Phys. Sci..

[cit20] Ahmad M. , Devereux R., Russel A. and Langton M. J., ChemRxiv, 2025, preprint, 10.26434/chemrxiv-2025-qtt7h

[cit21] Mitchell P. (1961). Nature.

[cit22] Murray C. J. L., Ikuta K. S., Sharara F., Swetschinski L., Robles Aguilar G., Gray A., Han C., Bisignano C., Rao P., Wool E. (2022). et al.. Lancet.

[cit23] Global antibiotic resistance surveillance report 2025, World Health Organization, 2025, https://www.who.int/publications/i/item/9789240116337

[cit24] McNaughton D. A., York E., Rawling T., Gale P. A. (2024). Org. Biomol. Chem..

[cit25] Hilton K. L. F., Manwani C., Boles J. E., White L. J., Ozturk S., Garrett M. D., Hiscock J. R. (2021). Chem. Sci..

[cit26] Maslowska-Jarzyna K., Rooijmans S., McNaughton D. A., Ryder W. G., York E., Tromp M., Gale P. A. (2025). Chem. Commun..

[cit27] Spooner M. J., Gale P. A. (2015). Chem. Commun..

[cit28] McNally B. A., Koulov A. V., Lambert T. N., Smith B. D., Joos J.-B., Sisson A. L., Clare J. P., Sgarlata V., Judd L. W., Magro G. (2008). et al.. Chem.–Eur. J..

[cit29] Torrisi J., Chvojka M., Jurček P., Zhang X., Zeng H., Šindelář V., Valkenier H. (2025). Angew. Chem., Int. Ed..

[cit30] Chowdhury S. M., Daood N. J., Lewis K. R., Salam R., Zhu H., Busschaert N. (2025). Digital Discovery.

[cit31] Knight N. J., Hernando E., Haynes C. J. E., Busschaert N., Clarke H. J., Takimoto K., Garcia-Valverde M., Frey J. G., Quesada R., Gale P. A. (2016). Chem. Sci..

[cit32] Busschaert N., Bradberry S. J., Wenzel M., Haynes C. J. E., Hiscock J. R., Kirby I. L., Karagiannidis L. E., Moore S. J., Wells N. J., Herniman J. (2013). et al.. Chem. Sci..

[cit33] Yang K., Lee L. C., Kotak H. A., Morton E. R., Chee S. M., Nguyen D. P. M., Keskküla A., Haynes C. J. E. (2025). Chem. Methods.

[cit34] Popoola P. I. A., Allam T. L., Lilley R. J., Manwani C., Keers O. B., Tan J., Yang K., Long Y., Clark E. R., White L. J., Hilton K. L. F., Rankin J., Baker J., Bennett C., Wilson H. B., Morton E. R., Keskküla A., Martin B., O’Connor C., Sutton J. M., Hind C. K., Garrett M. D., Haynes C. J. E., Hiscock J. R. (2026). Chem. Sci..

[cit35] Gilchrist A. M., Patrick W., Israel C.-B., Daniel A.-C., Xin W., Roberto Q., Gale P. A. (2021). Supramol. Chem..

[cit36] Maguire M. E., Cowan J. A. (2002). BioMetals.

[cit37] Roeßler M., Sewald X., Müller V. (2003). FEMS Microbiol. Lett..

[cit38] Melkikh A. V., Sutormina M. I. (2008). J. Theor. Biol..

[cit39] Park S.-H., Hwang I., McNaughton D. A., Kinross A. J., Howe E. N. W., He Q., Xiong S., Kilde M. D., Lynch V. M., Gale P. A. (2021). et al.. Chem.

[cit40] Klein B., Wörndl K., Lütz-Meindl U., Kerschbaum H. H. (2011). Apoptosis.

[cit41] Abdalah R., Wei L., Francis K., Yu S. P. (2006). Neurosci. Lett..

[cit42] Harold F. M., Baarda J. R. (1967). J. Bacteriol..

[cit43] Marques I., Costa P. M. R., Miranda M. Q., Busschaert N., Howe E. N. W., Clarke H. J., Haynes C. J. E., Kirby I. L., Rodilla A. M., Pérez-Tomás R. (2018). et al.. Phys. Chem. Chem. Phys..

[cit44] McNaughton D. A., Macreadie L. K., Gale P. A. (2021). Org. Biomol. Chem..

[cit45] Burns J. R., Seifert A., Fertig N., Howorka S. (2016). Nat. Nanotechnol..

[cit46] Moore S. J., Wenzel M., Light M. E., Morley R., Bradberry S. J., Gomez-Iglesias P., Soto-Cerrato V., Perez-Tomas R., Gale P. A. (2012). Chem. Sci..

[cit47] Yang K., Boles J. E., White L. J., Hilton K. L. F., Lai H. Y., Long Y., Hiscock J. R., Haynes C. J. E. (2022). RSC Adv..

[cit48] Simard J. R., Pillai B. K., Hamilton J. A. (2008). Biochemistry.

[cit49] Wu X., Gale P. A. (2016). J. Am. Chem. Soc..

[cit50] White L. J., Wells N. J., Blackholly L. R., Shepherd H. J., Wilson B., Bustone G. P., Runacres T. J., Hiscock J. R. (2017). Chem. Sci..

[cit51] White L. J., Tyuleva S. N., Wilson B., Shepherd H. J., Ng K. K. L., Holder S. J., Clark E. R., Hiscock J. R. (2018). Chem.–Eur. J..

[cit52] Hilton K. L. F., Steyn H. J. F., Luthuli K. S., Rice M., Streather B. R., Sweeney E., White L. J., Morgan F. R., Rankin J., Baker J. (2025). et al.. J. Mater. Chem. B.

[cit53] Steyn H. J. F., White L. J., Hilton K. L. F., Hiscock J. R., Pohl C. H. (2024). ACS Omega.

[cit54] Boles J. E., Williams G. T., Allen N., White L. J., Hilton K. L. F., Popoola P. I. A., Mulvihill D. P., Hiscock J. R. (2022). Adv. Ther..

[cit55] White L. J., Boles J. E., Allen N., Alesbrook L. S., Sutton J. M., Hind C. K., Hilton K. L. F., Blackholly L. R., Ellaby R. J., Williams G. T. (2020). et al.. J. Mater. Chem. B.

[cit56] Boles J. E., Bennett C., Baker J., Hilton K. L. F., Kotak H. A., Clark E. R., Long Y., White L. J., Lai H. Y., Hind C. K. (2022). et al.. Chem. Sci..

[cit57] Blackholly L. R., Shepherd H. J., Hiscock J. R. (2016). CrystEngComm.

[cit58] Allen N., White L. J., Boles J. E., Williams G. T., Chu D. F., Ellaby R. J., Shepherd H. J., Ng K. K. L., Blackholly L. R., Wilson B. (2020). et al.. ChemMedChem.

[cit59] Rutkauskaite A., White L. J., Hilton K. L. F., Picci G., Croucher L., Caltagirone C., Hiscock J. R. (2022). Org. Biomol. Chem..

[cit60] Rutkauskaite A., White L. J., Boles J. E., Hilton K. L. F., Clifford M., Patenall B., Streather B. R., Mulvihill D. P., Henry S. A., Shepherd M. (2021). et al.. Supramol. Chem..

[cit61] White L. J., Streather B., Sutton J. M., Rankin J., Baker J., Bennett C., Wilson H. B., Hind C. K., Hiscock J. R. (2025). Org. Biomol. Chem..

[cit62] DeMars Z., Singh V. K., Bose J. L. (2020). J. Bacteriol..

[cit63] *E. coli* Extract, Total Powder Avanti Polar Lipids, https://www.sigmaaldrich.com/GB/en/product/avanti/100500p, accessed 6th November 2025

[cit64] Andrews N. J., Haynes C. J. E., Light M. E., Moore S. J., Tong C. C., Davis J. T., Harrell W. A., Gale P. A. (2011). Chem. Sci..

[cit65] Hu J., Cochrane W. G., Jones A. X., Blackmond D. G., Paegel B. M. (2021). Nat. Chem..

[cit66] Mitchell P., Moyle J. (1967). Nature.

[cit67] Tyc O., Tomás-Menor L., Garbeva P., Barrajón-Catalán E., Micol V. (2016). PLoS One.

[cit68] Baker C. N., Banerjee S. N., Tenover F. C. (1994). J. Clin. Microbiol..

[cit69] Lozano-Chiu M., Lancaster M. V., Rex J. H. (1998). Diagn. Microbiol. Infect. Dis..

[cit70] Rampersad S. N. (2012). Sensors.

[cit71] WHO bacterial priority pathogens list, 2024: bacterial pathogens of public health importance to guide research, development and strategies to prevent and control antimicrobial resistance, World Health Organization, 2024, https://www.who.int/publications/i/item/9789240093461 10.1016/S1473-3099(25)00118-5 PMC12367593 40245910

[cit72] Delcour A. H. (2009). Biochim. Biophys. Acta, Proteins Proteomics.

[cit73] O'Shea R., Moser H. E. (2008). J. Med. Chem..

[cit74] Gironi B., Kahveci Z., McGill B., Lechner B.-D., Pagliara S., Metz J., Morresi A., Palombo F., Sassi P., Petrov P. G. (2020). Biophys. J..

[cit75] Nasr M. M., Pourmadadi M., Yazdian F., Rashedi H., Rahdar A., Aboudzadeh M. A. (2025). Part. Part. Syst. Charact..

[cit76] Alonso-Carrillo D., Carreira-Barral I., Mielczarek M., Sancho-Medina A., Herran E., Vairo C., Del Pozo A., Luzuriaga I., Lazcanoiturburu N., Ibarrola O. (2023). et al.. Org. Biomol. Chem..

[cit77] Medina-Carmona E., Varela L., Hendry A. C., Thompson G. S., White L. J., Boles J. E., Hiscock J. R., Ortega-Roldan J. L. (2020). Chem. Commun..

[cit78] Strahl H., Errington J. (2017). Annu. Rev. Microbiol..

[cit79] Automated script, https://github.com/ta1u18/EC50-high-throughput-workflow

