# Supplementary material for: An increased throughput workflow to identify ion transport and membrane lysis agents for antimicrobial discovery
Source: Chem Sci. 2026 Mar 3;17(17):8580–94. doi: 10.1039/d5sc09781a (PMC12970626; doi:10.1039/d5sc09781a)
Supplement: SC-017-D5SC09781A-s001 [file SC-017-D5SC09781A-s001.pdf]

Supplementary Information (SI) for Chemical Science.
This journal is © The Royal Society of Chemistry 2026

Supporting Information

An increased throughput workflow to assess biologically relevant ion transport and membrane lysis for antimicrobial discovery

Kylie Yang,[a, b] Caleb Marsh,[c] Lisa J. White,[d] Fergus W. Molyneux,[a] Thomas L. Allam,[e] Precious I. A. Popoola,[d] Olivia B. Keers,[d] Matthew Rice,[d] Kira L. F. Hilton,[d] Hiral A. Kotak,[a] J. Mark Sutton,[c] Jose L. Ortega-Roldan,[d] Charlotte K. Hind,[c]* Jennifer R. Hiscock[d]* and Cally J. E. Haynes[a]*

[a] Department of Chemistry, University College London, 20 Gordon Street, London WC1H, 0AJ, UK. Email: cally.haynes@ucl.ac.uk.

[b] School of Physics, Chemistry and Earth Sciences, University of Adelaide, Adelaide, SA 5005, Australia.

[c] Research and Evaluation, Porton Down, UKHSA, Porton Down, Salisbury SP4 0JG, UK. Email: Charlotte.Hind@UKHSA.gov.uk.

[d] School of Natural Sciences, University of Kent, Canterbury, CT2 7NH, UK. E-mail: J.R.Hiscock@Kent.ac.uk.

[e] School of Chemistry, University of Southampton, Highfield, Southampton, SO17 1BJ, UK.

S1 Synthesis

S1.1 Previously reported compounds.

The following chemical compounds investigated for their ion transport activities were obtained from the chemical libraries contained within the synthetic labs of Hiscock and Haynes. The synthesis and full characterisation of these compounds has been supplied within the scope of the publications listed.

AT full chemical characterisation available in reference [1].

Compound **2:** full chemical characterisation available in reference [2].

Compound **4:** full chemical characterisation available in reference [3].

Compound **5:** full chemical characterisation available in reference [2].

Compound **6:** full chemical characterisation available in reference [2].

Compound **8:** full chemical characterisation available in reference [3].

Compound **15:** full chemical characterisation available in reference [3].

Compound **27:** full chemical characterisation available in reference [3].

Compound **28:** full chemical characterisation available in reference [2].

Compound **29:** full chemical characterisation available in reference [2].

Compound **30:** full chemical characterisation available in reference [2].

Compound **38:** full chemical characterisation available in reference [4].

Compound **39:** full chemical characterisation available in reference [4].

Compound **40:** full chemical characterisation available in reference [5].

Compound **43:** full chemical characterisation available in reference [5].

Compound **47:** full chemical characterisation available in reference [6].

Compound **48:** full chemical characterisation available in reference [7].

Compound **50:** full chemical characterisation available in reference [7].

Compound **51:** full chemical characterisation available in reference [7].

Compound **56:** full chemical characterisation available in reference [8].

Compound **57:** full chemical characterisation available in reference [8].

Compound **61:** full chemical characterisation available in reference [7].

Compound **69:** full chemical characterisation available in reference [9].

Compound **72:** full chemical characterisation available in reference [8].

Compound **73:** full chemical characterisation available in reference [8].

Compound **74:** full chemical characterisation available in reference [10].

Compound **75:** full chemical characterisation available in reference [10].

Compound **86:** full chemical characterisation available in reference [9].

Compound **93:** full chemical characterisation available in reference [11].

Compound **94:** full chemical characterisation available in reference [11].

Compound **101:** full chemical characterisation available in reference [11].

Compound **102:** full chemical characterisation available in reference [11].

Compound **103:** full chemical characterisation available in reference [11].

Compound **104:** full chemical characterisation available in reference [11].

Compound **109:** full chemical characterisation available in reference [11].

Compound **110:** full chemical characterisation available in reference [11].

Compound **111:** full chemical characterisation available in reference [11].

Compound **112:** full chemical characterisation available in reference [11].

Compound **113:** full chemical characterisation available in reference [11].

Compound **114:** full chemical characterisation available in reference [11].

S1.2 Synthesis of novel compounds: general remarks

A positive pressure of nitrogen and oven dried glassware were used for all reactions. All solvents and starting materials were purchased from known chemical suppliers or available stores and used without further purification. The melting point for each compound was measured using a Stuart SMP10 melting point apparatus. High resolution mass spectrometry was performed using a Brucker microTOF-Q mass spectrometer and spectra recorded and processed using Brucker's Compass Data Analysis software. Infrared spectra were obtained using Shimadzu IR-Affinity-1 model Infrared spectrometer. The data was analysed in wavenumbers (cm^{-1}) using IRsolution software.

S1.3 Synthetic protocols

Compound 97

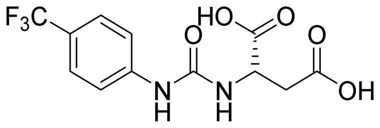

1-Isocyanato-4-(trifluoromethyl) benzene (0.57 mL, 4.00 mmol) was added to L-aspartic acid dimethyl ester hydrochloride (0.83 g, 4.20 mmol) and triethylamine

(0.72 mL, 5.20 mmol) in acetonitrile (20 mL) and stirred at room temperature overnight. The mixture was then taken to complete dryness and dissolved in chloroform (5 mL), followed by dropwise additions of hexane (> 5 mL) resulting in precipitation. The precipitate was collected via filtration. The precipitate was dissolved in isopropanol (5 mL) and sodium hydroxide (2 mL, 2M) and stirred at room temperature for 4 hours. Hydrochloric acid (1M) was added dropwise until precipitation (~pH 5) and collected by filtration. The pure product was identified using NMR spectroscopy and collected as a white solid with a yield of 33 % (0.426 g, 1.33 mmol); melting point: > 200 °C; ^{1}H NMR (400 MHz, 298 K, DMSO-d$_6$): δ: 12.59 (bs, 2H), 9.28 (s, 1H), 7.58 (s, 4H), 6.71 - 6.69 (d, J = 8.36 Hz, 1H), 4.54 – 4.50 (q, J = 5.04 Hz, 2H), 2.84 – 2.83 (m, 2H); ^{13}C{^{1}H} NMR (100 MHz, 298 K, DMSO-d$_6$): δ: 173.3 (CO), 172.7 (CO), 154.8 (CO), 144.4 (ArC), 126.5 (ArCH), 129.1 – 121.0 (q, J = 268.7 Hz, ArC), 122.0 – 121.2 (q, J = 31.83 Hz, CF_3), 117.7 (ArCH), 49.2 (CH), 37.1 (CH_2); IR (film): v = 3001 (NH stretch), 1701, 1414, 1304, 844; HRMS for the carboxylic acid-urea ($C_{12}H_{11}F_3N_2O_5$) (ESI$^-$): m/z: act = 320.0620 [M]$^-$, cal = 320.0668 [M]$^-$.

Compound 99

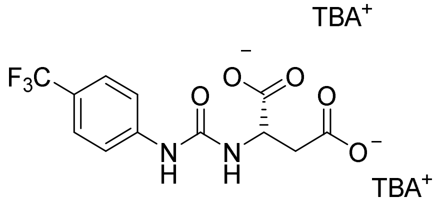

Tetrabutylammonium hydroxide (1.50 mL, 1.50 mmol) was added to **97** (0.50 g, 1.50 mmol) in methanol. The mixture was then taken to complete dryness. The pure product was identified using NMR spectroscopy and collected as a clear oil with a yield of 100 % (1.20 g, 1.50 mmol); melting point: oil; ^{1}H NMR (400 MHz, 343 K, DMSO-d_6): δ: 12.91 (bs, 1H), 7.71 (s, 1H), 7.47 – 7.45 (d, J = 7.08 Hz, 2H), 6.14 (s, 1H), 3.81 (s, 1H), 3.22 – 3.17 (m, 16H), 2.61 (s, 1H), 1.94 (s, 1H), 1.62 – 1.60 (m, 16H), 1.40 – 1.32 (m, 16H), 0.98 – 0.94 (m, 24H); ^{13}C{^{1}H} NMR (100 MHz, 298 K, DMSO-d_6): δ: 176.5 (CO), 173.8 (CO), 157.4 (CO), 146.5 (ArC), 129.4 – 121.3 (q, J = 261.9 Hz, ArC), 125.8 (ArCH), 120.9 – 120.3 (q, J = 25.2 Hz, CF_3), 118.6 (ArCH), 58.1 (CH_2), 56.0 (CH), 46.8 (CH_2), 23.6 (CH_2), 19.6 (CH_2), 14.0 (CH_3); IR (film): v = 2995 (NH stretch), 1669, 1437, 1300, 874; HRMS for the carboxylate-urea ($C_{12}H_9F_3N_2O_5^{2-}$) (ESI$^-$): m/z: act = 318.0475 [M]$^-$, cal = 318.0401 [M]$^-$.

S1.4 NMR Spectra

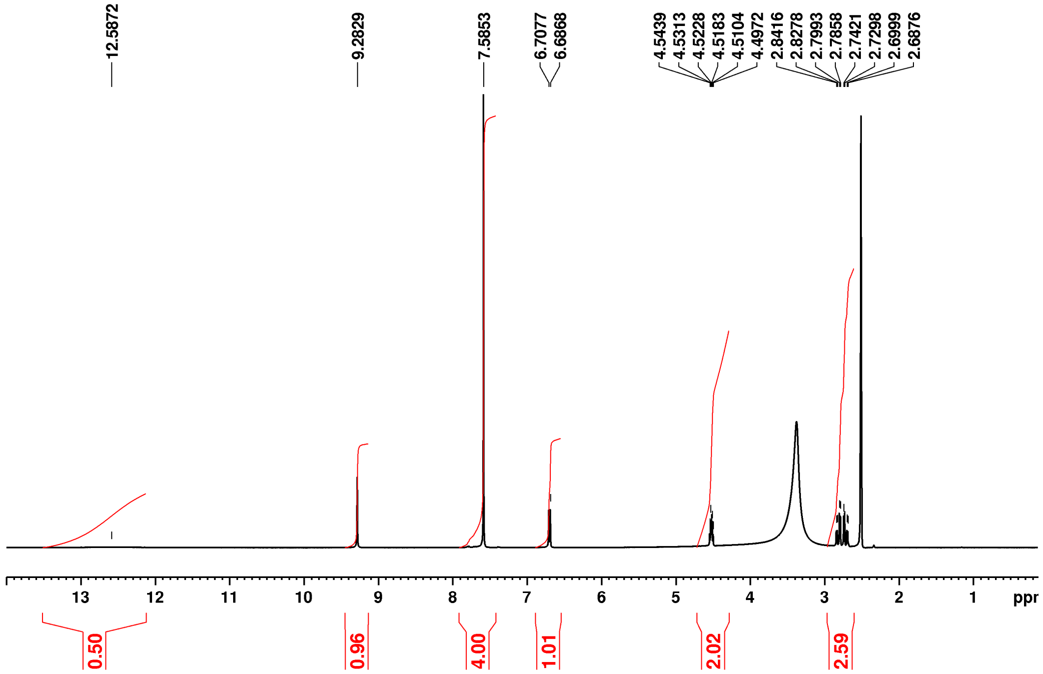

*Figure S 1 ^{1}H NMR spectrum of **97** in DMSO-d_6 conducted at 298 K.*

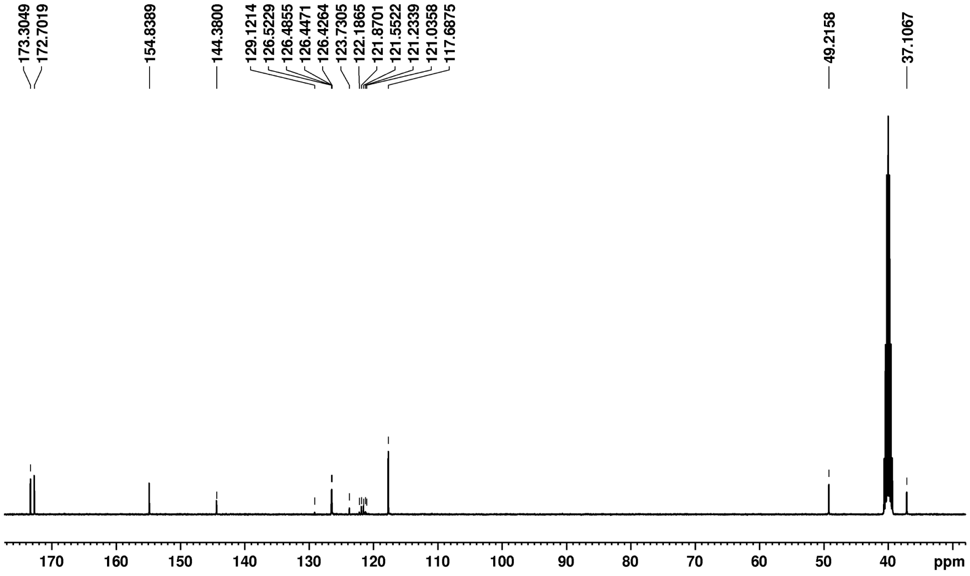

*Figure S 2 $^{13}C\{^{1}H\}$ NMR spectrum of **97** in DMSO-d_6 conducted at 298 K.*

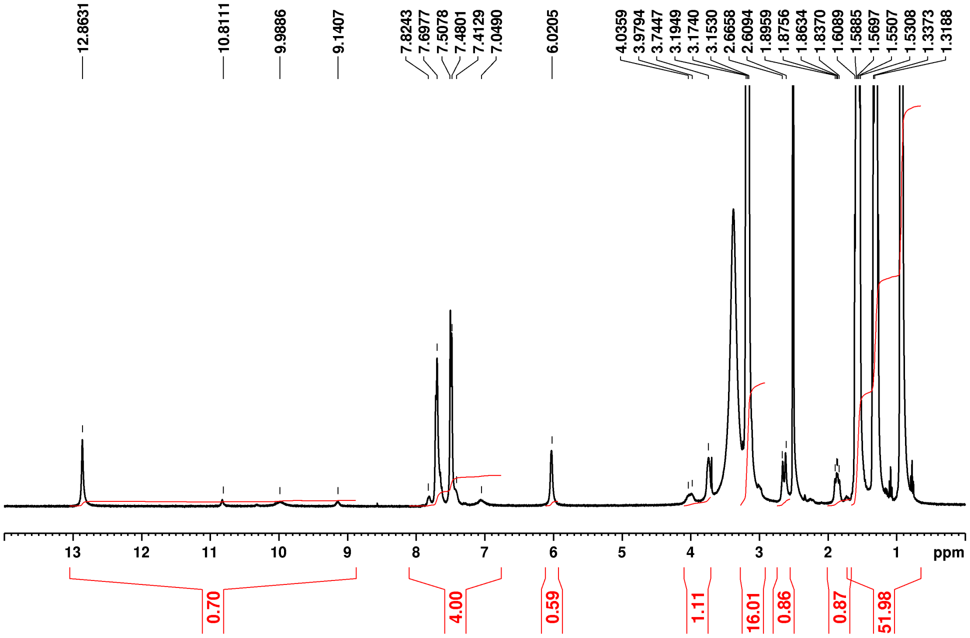

Figure S 3 ^{1}H NMR spectrum of **99** in DMSO-d_6 conducted at 298 K.

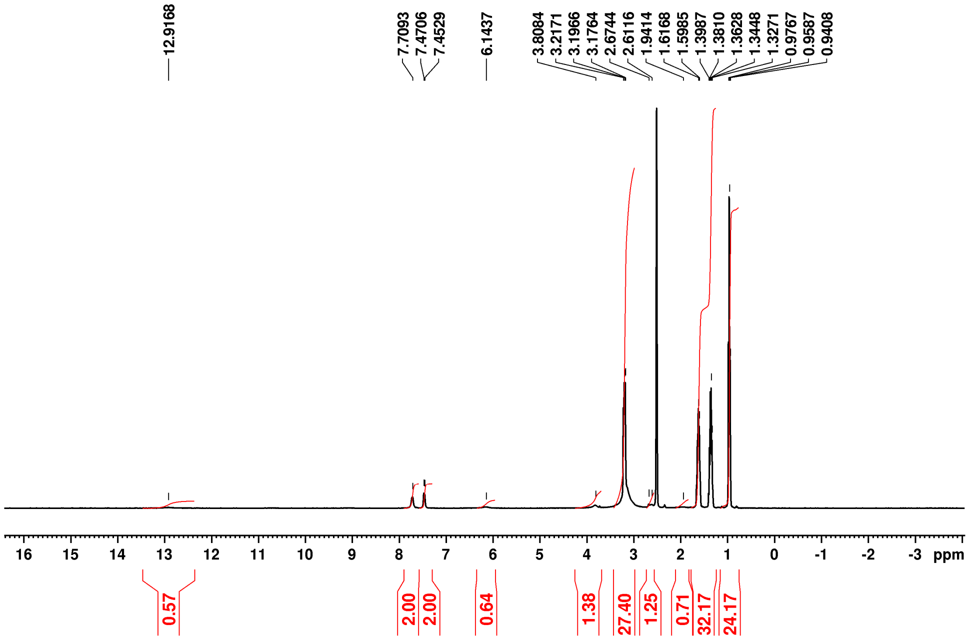

Figure S 4 ^{1}H NMR spectrum of **99** in DMSO-d_6 conducted at 343 K.

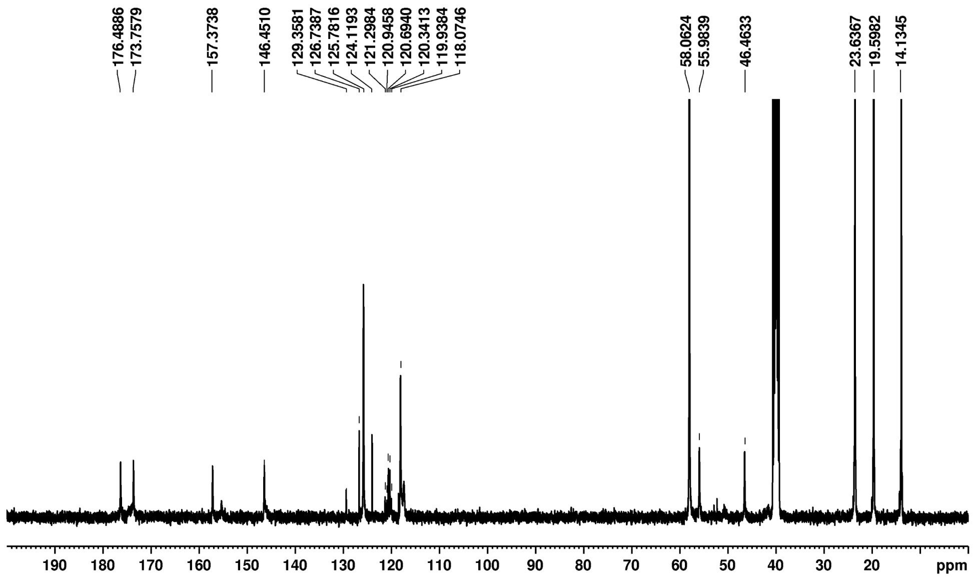

*Figure S 5 $^{13}C\{^{1}H\}$ NMR spectrum of **99** in DMSO-d_6 conducted at 298 K.*

S2 Ion transport protocols

S2.1 Preparation of dye-loaded vesicles

A stock solution of lipid was prepared in de-acidified chloroform at a concentration of 1 g per 35 mL. The three lipids used in this study are; 1-palmitoyl-2-oleoyl-sn-glycero-3-phosphocholine (POPC, purchased from CliniSciences or Avanti Polar Lipids (Merck)), 1-palmitoyl-2-oleoyl-sn-glycero-3-phosphoethanolamine (POPE, purchased from purchased from CliniSciences or Avanti Polar Lipids (Merck)) and 1-Palmitoyl-2-oleoyl-sn-glycero-3-(phospho-rac-(1-glycerol)) (POPG purchased from purchased from CliniSciences or Avanti Polar Lipids (Merck)). The stock solution was stored in an air-tight brown glass container at -20 °C. For a typical transport experiment, 1.0 mL of the lipid solution was transferred to a round-bottom flask, and the chloroform was removed on a rotary evaporator, and the flask was left on vacuum for 2 hours. The lipid film was then hydrated with a solution of dye in buffer using a volume equal to the quantity of lipid stock solution used. The dyes used in this study are; HPTS (1 mM) dissolved in either potassium chloride (100 mM, 10 mM HEPES, pH = 7.0), potassium gluconate (100 mM, 10 mM HEPES, pH = 7.0), or NMDG chloride (100 mM, 10 mM HEPES, pH = 7.0), and Sulforhodamine B (50 mM) dissolved in potassium chloride (75mM, 10mM HEPES, pH = 7.0). The sample was sonicated at 40 °C until all lipid was suspended. The lipid sample was then subjected to 9 freeze-thaw cycles. At the end of the freeze-thaw process, the sample can be used immediately or stored in the freezer for no more than 72 hours. After thawing, the lipid was allowed to rest for 30 minutes and then extruded a minimum of 25 times through 200 nm polycarbonate membranes using an Avanti mini-extruder. The vesicles were purified by size exclusion chromatography on Sephadex G50, eluting with the appropriate buffer (potassium chloride, potassium gluconate or NMDG chloride, 100 mM, 10 mM HEPES buffer, pH = 7.0). The size exclusion chromatography was performed to remove any un-encapsulated dye, and the vesicles were used the same day.

S2.2 High-throughput transport experiments

Transporter stock solutions were added to a solvent-resistant polypropylene 96-well plate (Costar). Additional controls included the solvent (DMSO or 5% ethanol in water), known transporters (valinomycin + CCCP or AT dissolved in DMSO or EtOH as appropriate) and triton (10% w/v). For Hill plot analyses, different concentrations of transporters were prepared within the same drug plate by serial dilution.

HPTS-loaded vesicles were prepared according to section S2.1**.** The vesicle solution (150 μL, 2×, 0.2 - 2 mM) was manually added to each well of a 96 well plate (Thermofisher, black, flat bottom). The transporter stock solution was manually added and mixed with the vesicles using a multichannel pipette. The same volume of transporter (or control sample) was added to each well.

Basic buffer was prepared (at least 20 mL per plate) to drop the external pH to 8, marking the beginning of the the assay. These buffers include: potassium chloride buffer (17.71 mL, 100 mM with 10 mM HEPES, pH = 7.0) with KOH (2.27 mL, 100 mM), potassium gluconate buffer (17.47 mL, 100 mM with 10 mM HEPES, pH = 7.0) with KOH (2.53 mL, 100 mM), and NMDG chloride buffer (16.80 mL, 100 mM with 10 mM HEPES, pH = 7.0) with NMDG base (3.20 mL, 100 mM).

The assay plate was analysed on a plate reader (Tecan Spark fitted with an autoinjector). The method file (provided as an additional Supplementary Information file) involves addition of the basic buffer (150 μL) via the autoinjector to each well. Each well is scanned precisely 5 minutes after addition of the base at two excitation wavelengths (λ_{ex} = 403, λ_{em} = 510 and λ_{ex} = 460 nm, λ_{em} = 510 nm).

S2.3 HPTS data processing

Each plate contained at least 3 wells for a negative control (typically blank solvent – used to calibrate 0% efflux) and at least 3 wells for positive controls (known transporters – used to calibrate 100% efflux).

The ratiometric fluorescence response of the HPTS in each well was calculated as

$$R = \frac{I_{460}}{I_{403}}$$

Where I_{460} and I_{403} are the emission intensity of HPTS at 510 nm after excitation at 460 nm and 403 nm respectively.

The percentage efflux was calculated according to the following equation:

$$\%\, efflux = \frac{R - R_0}{R_{100} - R_0}$$

Where R_0 = the mean ratiometric response of the negative control wells and R_{100} = the mean ratiometric response of the positive control wells.

S2.4 Hill plot analysis

Hill plot data was analysed using OriginLab (2019) software, and fitted to the Hill1 equation:

$$y = V_{min} + (V_{max} - V_{min})\frac{x^n}{k^n + x^n}$$

Where:

x = the transporter concentration;

y = the response (% efflux);

V_{min} = the minimum observed response;

V_{max} = the maximum response, fixed here at 100% efflux;

k = EC_{50} ; and

n = the Hill coefficient.

S2.5 Sulforhodamine B (SRB) release assay

Transporter stock solutions were prepared in a solvent-resistant polypropylene 96-well plate (Costar). Additional controls included the solvent (DMSO or 5% ethanol in water), and Triton (10% w/v).

Vesicles encapsulating Sulforhodamine B were prepared according to section S2.1. The vesicle solution (150 µL, 2×, 2 – 0.2 mM lipid) was added to each well of a 96 well plate (Thermofisher, black, flat bottom). The transporter stock solution was added and mixed with the vesicles using a multichannel pipette, ensuring the same volume was added to each well. The plate was left for a minimum of 1 hour for the lipid and transporter to interact, then the assay plate was analysed on a plate reader (λ_{ex} = 559 nm, λ_{em} = 586 nm). An electronic method file for this process is provided as additional Supplementary Information.

The percentage of lysis was calculated according to the following equation:

$$\%\ Lysis = \frac{F - F_0}{F_{100} - F_0}$$

Where F = the fluorescence emission intensity of the well in question, F_0 = the mean fluorescence emission intensity of the negative control wells and F_{100} = the mean fluorescence emission intensity of the positive control wells.

S3 Ion transport data

S3.1 Benchmarking compounds

Table S1 Observed assay results for known transporters in different buffers. Values represent the change in the fluorescence ratio (%) normalised with respect to DMSO (0%) and a known active transporter (100%). The known transporter was chosen to be valinomycin + CCCP in buffers that contain potassium, or prodigiosin in buffers that contain chloride. The concentrations tested were 0.00059% for prodigiosin, 0.1% for CCCP, 0.59% for AT, 0.1% for monensin, 0.1% for valinomycin. Lipid concentration = 0.1 mM.

	KCl	KCl + CCCP	K glu	K Gluconate + CCCP	NMDG chloride	NMDG chloride + CCCP
**CCCP**	2 ± 1		3 ± 0.4		1.6 ± 0.6	
**DMSO**	0 ± 1		0 ± 1		0 ± 0.6	
**Valinomycin**	-5 ± 0.6	100 ± 3	1 ± 0.9	100 ± 5	-3 ± 0.7	-7 ± 1
**Monensin**	101 ± 2	92 ± 1	100 ± 1	96 ± 3	-19 ± 2	-23 ± 1
**AT**	102 ± 0.3	102 ± 5	21 ± 0.2	32 ± 0.4	100 ± 1	100 ± 2
**Prodigiosin**	100 ± 2	104 ± 2	17 ± 0.5	32 ± 0.3	100 ± 2	101 ± 0.3

S3.2 Library 1

Table S2 Assay results for Library 1 compounds tested at 10 mol% with respect to POPC. Values represent the change in the fluorescence ratio (%) normalised with respect to DMSO (0%) and a known active transporter (100%). The known transporter was chosen to be valinomycin + CCCP in buffers that contain potassium, or prodigiosin in buffers that contain chloride. Lipid concentration = 0.1 mM.

	KCl	KCl + CCCP	KGlu	KGlu + CCCP	NMDG-Cl	NMDG-Cl + CCCP
**2**	10	4	10	3	3	2
**4**	29	27	14	5	16	23
**5**	1	-5	0	-2	0	-2
**6**	97	99	42	36	104	104
**8**	99	103	46	38	102	100
**15**	2	-3	0	-3	0	1
**27**	96	96	48	43	65	73
**28**	101	100	43	34	97	103

**29**	100	101	41	30	101	99
**38**	2	-5	3	-1	0	1
**39**	2	-4	4	-1	-4	-3
**40**	1	-6	1	-2	-1	0
**43**	9	4	4	0	2	2
**47**	1	-6	3	-3	-3	-4
**48**	1	-5	3	-6	-2	-2
**60**	2	-5	-1	-11	-10	-11
**51**	30	21	33	25	22	20
**61**	64	62	7	0	-5	-8
**69**	5	-3	2	-1	1	0
**74**	2	-5	2	-3	3	2
**75**	10	-2	5	0	19	24
**86**	36	28	48	41	23	17
**93**	34	33	17	6	10	12
**94**	83	86	30	19	57	66
**97**	26	21	16	4	6	7
**99**	18	17	13	5	4	5
**101**	71	73	28	13	53	42
**103**	3	-3	3	-1	2	-1
**109**	3	-5	1	-3	0	0
**111**	3	-3	3	0	1	-1
**113**	3	-5	0	-3	1	0

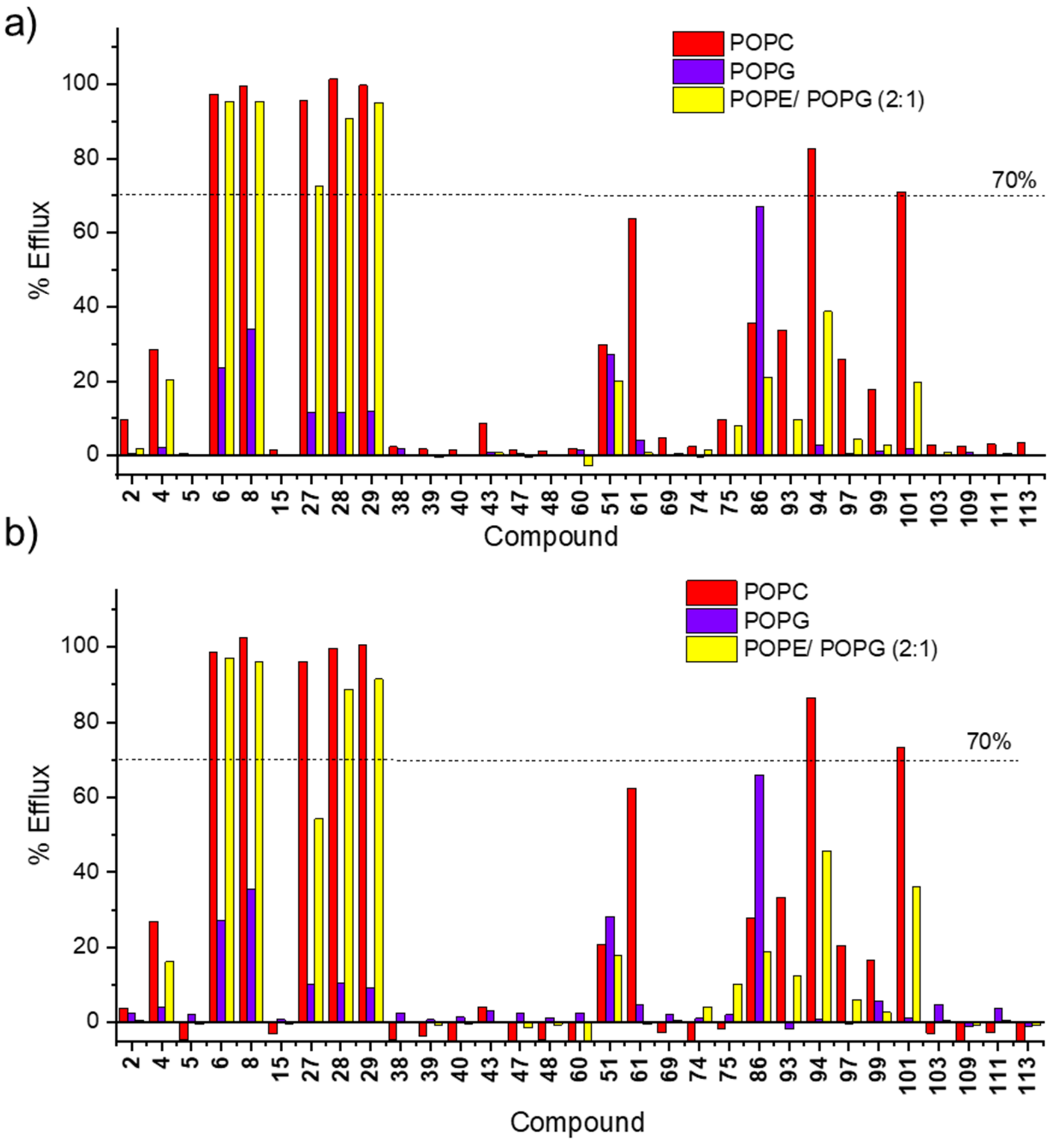

Figure S 6 KCl screening results for Library 1 (10 mol% w.r.t. lipid) in POPC and POPE/ POPG (2:1) vesicles: a) library compounds alone; b) library compounds + 0.1 mol% CCCP. Lipid concentration = 0.1 mM.

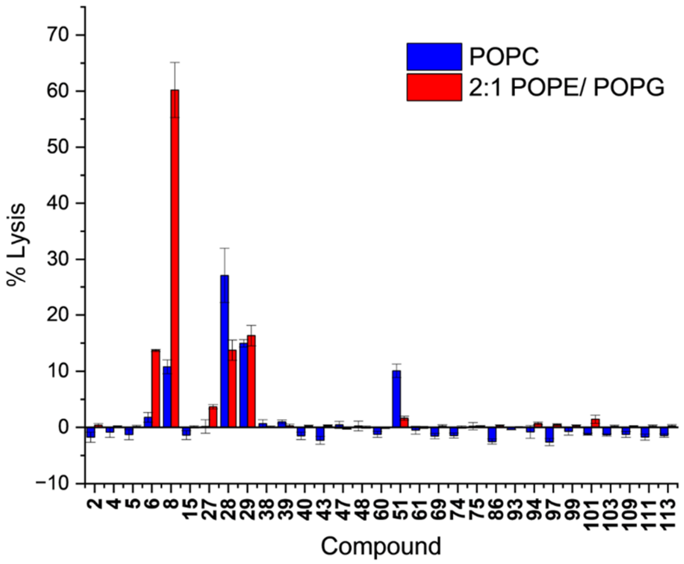

Figure S 7 SRB assay results for Library 1 (10 mol% w.r.t. lipid) in POPC and POPE/ POPG (2:1) vesicles. Lipid concentration = 0.1 mM.

Table S3 Assay results for active compounds in Library 1 at 10 mol% with respect to lipid in POPE/ POPG (2:1) vesicles. Values represent the change in the fluorescence ratio (%) normalised with respect to DMSO (0%) and a known active transporter (100%). The known transporter was chosen to be valinomycin + CCCP in buffers that contain potassium, or prodigiosin in buffers that contain chloride. Lipid concentration = 0.1 mM.

	**KGlu**	**NMDG-Cl**
**6**	29	27
**8**	20	98
**27**	8	11
**28**	15	32
**29**	10	49
**94**	3	15
**101**	0.5	5

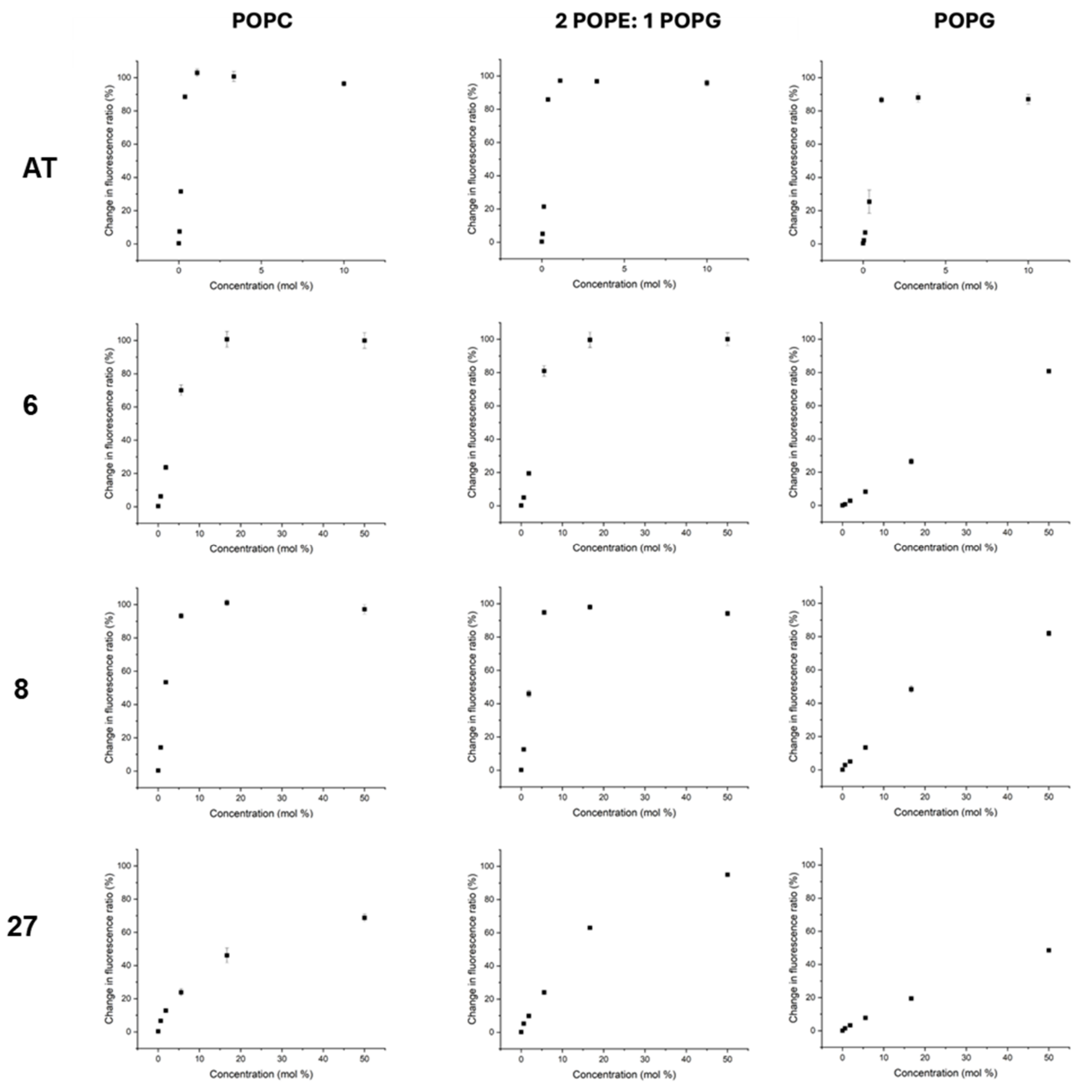

Figure S 8 Rough KCl dose-response plots conducted in three types of vesicles composed of different lipids. Data points represent three repeats and the error bar represents the standard deviation. Lipid concentration = 0.1 mM.

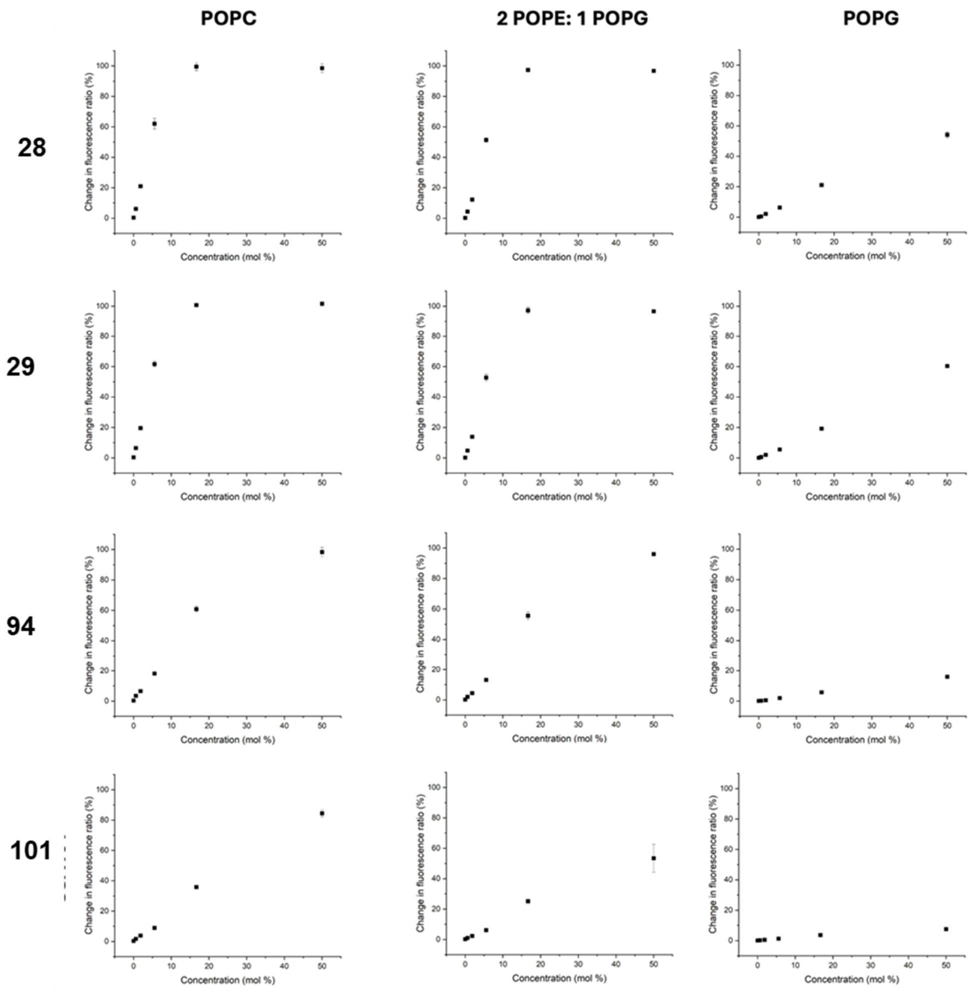

Figure S 9 Rough KCl dose-response plots conducted in three types of vesicles composed of different lipids. Data points represent three repeats and the error bar represents the standard deviation. Lipid concentration = 0.1 mM.

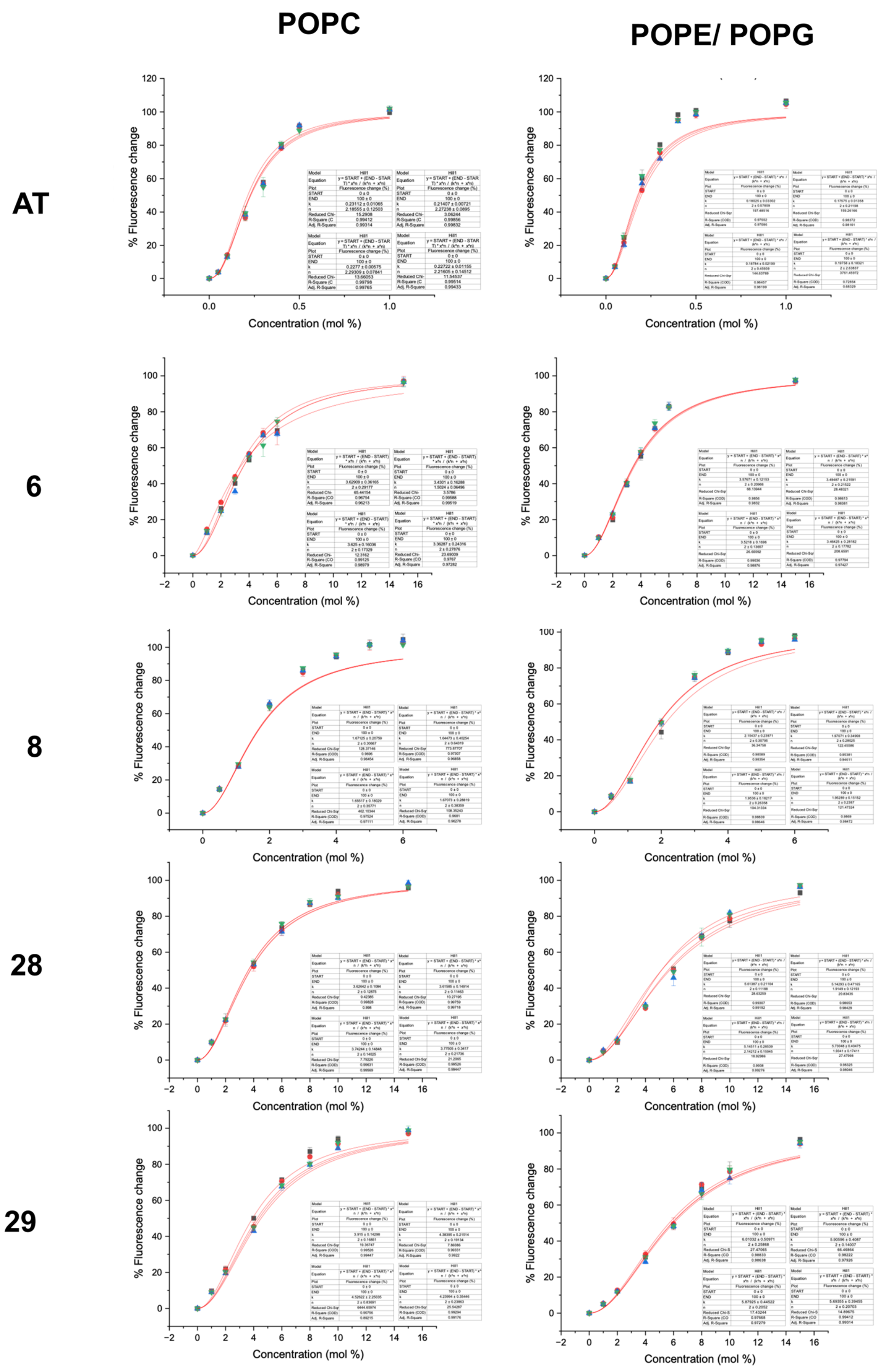

Figure S 10 Replicate Hill plots conducted in two types of vesicles composed of different compositions. Data points represent three the mean of repeats and error bars represent the standard deviation. Lipid concentration = 0.1 mM.

S3.3 Library 2

Experiments in this section were carried out at final lipid concentration of 1 mM lipid for better comparability to the previously obtained results.

Hill plot analysis

Lipid	56	57	56/57
POPC	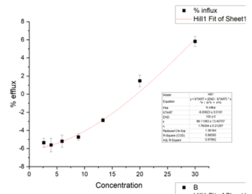	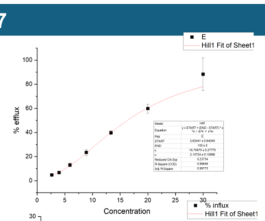	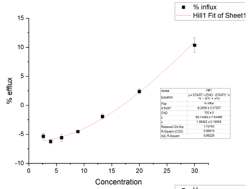
POPG	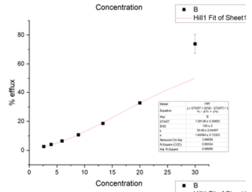	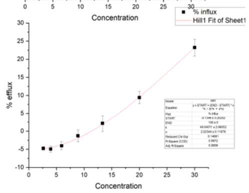	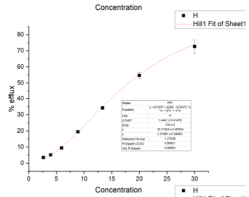
POPE/ POPG	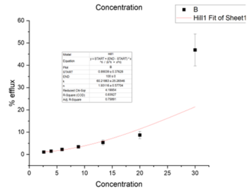	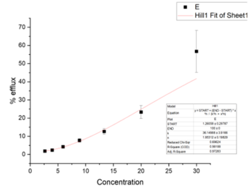	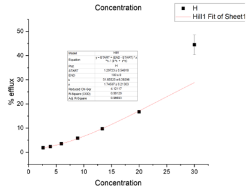

*Figure S 11 KCl Hill plot analysis for transporters **56**, **57** and a racemic mixture of **56**/ **57** in POPC, POPG and a 2:1 mixture of POPE/ POPG. Concentrations of transporter are given in mol% with respect to the lipid concentration. Each data point represents the mean of a measurements from a minimum of 3 individual wells, and error bars represent the standard deviation. Processing was carried out using Microsoft Excel, with fitting to the Hill1 equation performed in Origin 2019.*

Lipid	56	57	56/57
POPC	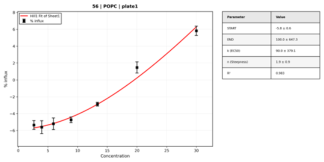	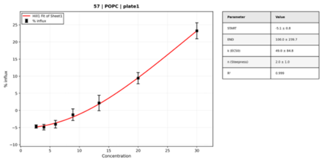	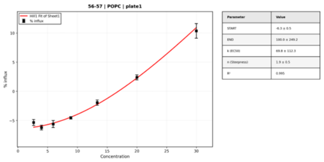
POPG	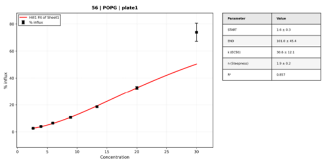	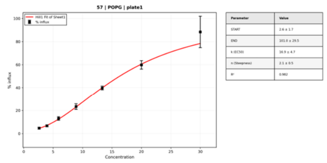	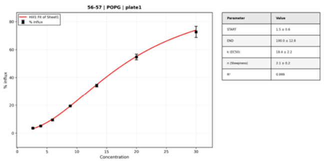
POPE/ POPG	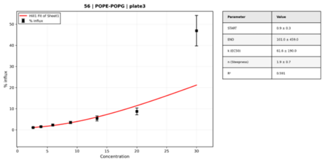	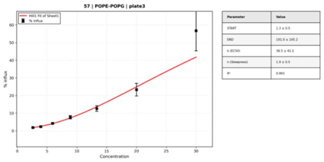	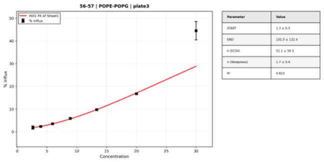

*Figure S 12 KCl Hill plot analysis for transporters **56**, **57** and a racemic mixture of **56**/ **57** in POPC, POPG and a 2:1 mixture of POPE/ POPG. Concentrations of transporter are given in mol% with respect to the lipid concentration. Each data point represents the mean of a measurements from a minimum of 3 individual wells, and error bars represent the standard deviation. Data processing and fitting was carried out using the Python script described in Section S7.*

Lipid	72	73	72/73
POPC	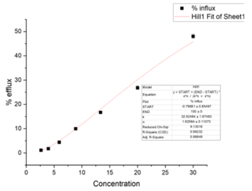	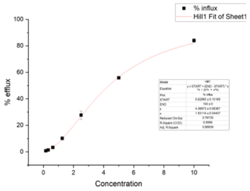	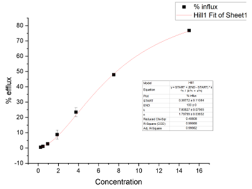
POPG	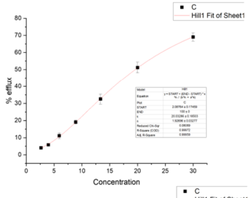	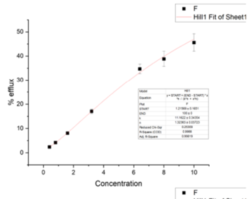	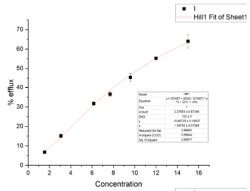
POPE/<br>POPG	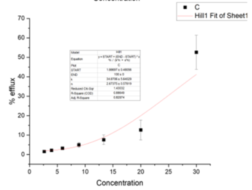	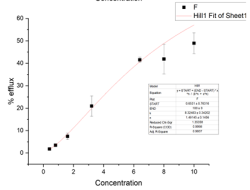	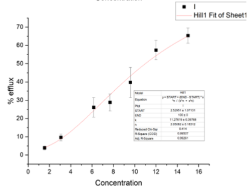

*Figure S 13 KCl Hill plot analysis for transporters **72**, **73** and a racemic mixture of **72**/ **73** in POPC, POPG and a 2:1 mixture of POPE/ POPG. Concentrations of transporter are given in mol% with respect to the lipid concentration. Each data point represents the mean of a measurements from a minimum of 3 individual wells, and error bars represent the standard deviation. Processing was carried out using Microsoft Excel, with fitting to the Hill1 equation performed in Origin 2019.*

Lipid	72	73	72/73
POPC	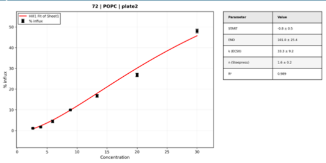	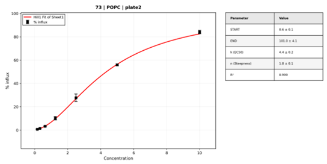	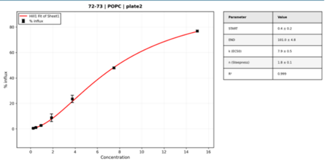
POPG	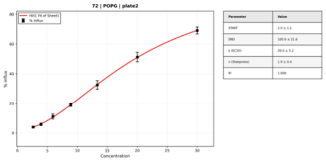	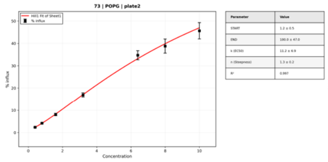	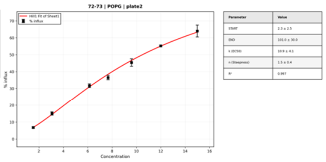
POPE/ POPG	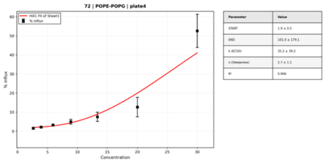	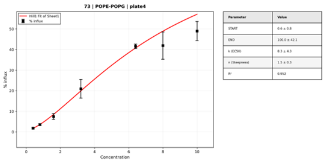	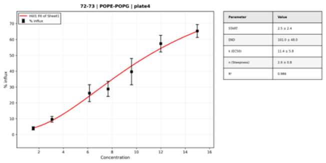

*Figure S 14 KCl Hill plot analysis for transporters **72**, **73** and a racemic mixture of **72**/ **73** in POPC, POPG and a 2:1 mixture of POPE/ POPG. Concentrations of transporter are given in mol% with respect to the lipid concentration. Each data point represents the mean of a measurements from a minimum of 3 individual wells, and error bars represent the standard deviation. Data processing and fitting was carried out using the Python script described in Section S7.*

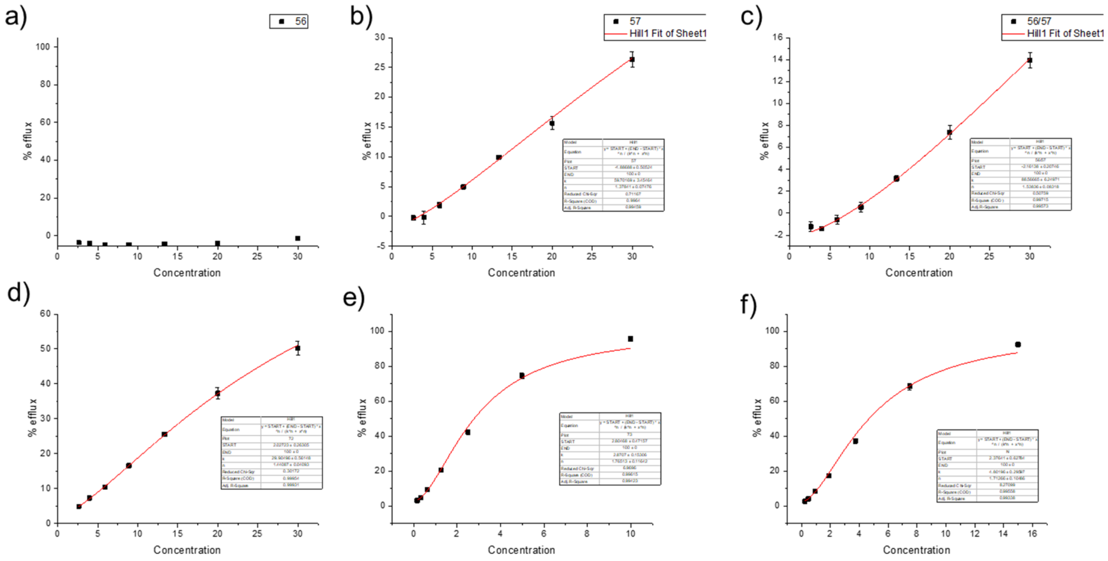

*Figure S 15 Dose response data and attempted Hill plot analysis in the KCl assay in POPC vesicles, using DMSO as a delivery solvent: a) compound **56**; b) compound **57**; c) a racemic mixture of **56**/ **57**; d) compound **72**; e) compounds **73**; f) a racemic mixture of **72**/ **73**. Concentrations of transporter are given in mol% with respect to the lipid concentration. Each data point represents the mean of a measurements from a minimum of 3 individual wells, and error bars represent the standard deviation. Processing was carried out using Microsoft Excel, with fitting to the Hill1 equation performed in Origin 2019.*

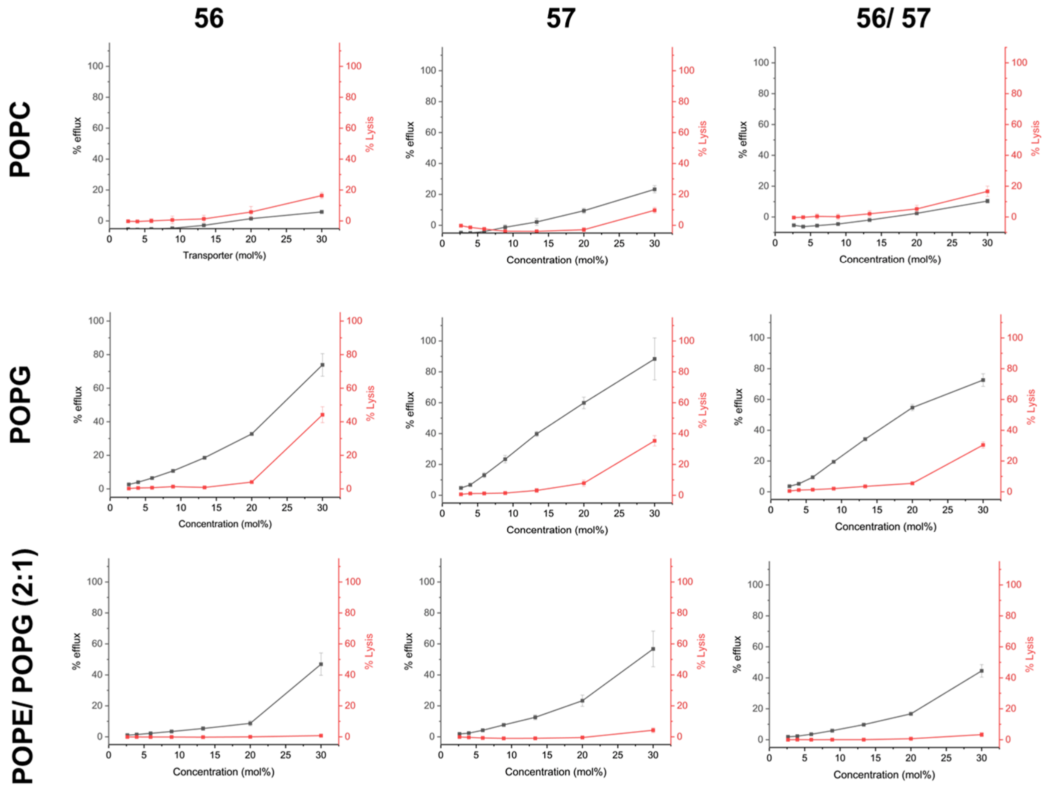

*Figure S 16 A comparison of dose-dependent transport (in the KCl assay) vs lysis (in the SRB assay) in different lipid mixtures for compounds **56**, **57** and a racemic mixture of **56**/ **57**.*

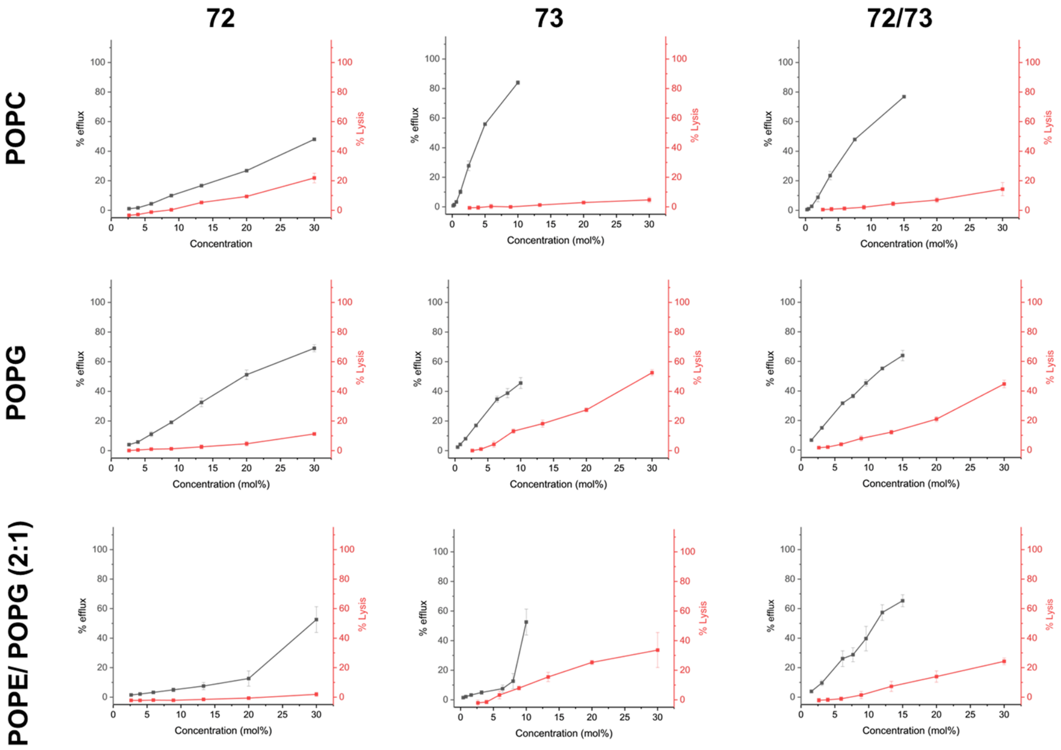

*Figure S 17 A comparison of dose-dependent transport (in the KCl assay) vs lysis (in the SRB assay) in different lipid mixtures for compounds **72**, **73** and a racemic mixture of **72**/ **73**.*

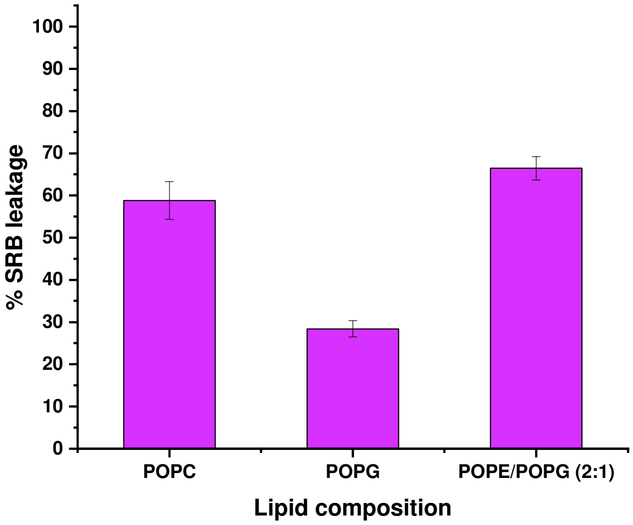

*Figure S 18 SRB leakage mediated by **AT** (1 mol% in EtOH) from vesicles composed of three different lipids/ mixtures.*

S4 Antimicrobial protocols and data

S4.1 Culture of bacteria

Bacterial strains were resuscitated from glycerol stocks stored at -80°C on tryptic soy agar (TSA) plates. These plates were stored at 4°C for a maximum of two weeks before passaging and passaged once before disposal. Aliquots of 3 mL of tryptic soy broth (TSB) in 30 mL universal tubes were inoculated with a 1 µL loopful of bacteria and incubated at 37°C and 200 rpm for a minimum of 18 hours.

S4.2 MIC determination

Aliquots of 100 µL of bacteria culture were suspended in 900 µL of TSB and the OD600 of the overnight culture was determined. Inoculum of 0.011 OD were prepared from the overnight culture in TSB and 190 µL aliquots were added to the desired wells of a polystyrene flat bottom 96 well plate (Greiner, 655182), the assay plate. Working stocks of compounds were prepared by adding the appropriate volume of DMSO (Merck) to suspend compounds at (50 mM). The compound plate was prepared by diluting these working stocks in DMSO across a flat bottom polypropylene 96 well plate (Greiner, 655201). The final column on the compound plate contained no compound. Aliquots of SSAs were taken from the compound plate, with 10 µL of the desired SSA added to the appropriate wells in the assay plate. Resulting in a final DMSO concentration of 10%. An additional assay plate was prepared containing TSB with no inoculum. Assay plates were loaded into a BMG Clariostar plus with stacker attached inside a 37°C incubator. The OD600 was measured for each assay plate every hour, for 20 hours. The OD600 at 20 hours was used to determine the MIC, with modal values reported.

S4.3 Inverted vs conventional MIC assay results

Comparison of MIC values determined by “Inverted” protocol (in which compounds were diluted in DMSO then added to wells containing buffer and bacteria), vs our “Conventional” protocol, in which compounds were diluted in DMSO and TSB before introducing bacteria. The results are shown in Table S4.

*Table S4 Comparable modal MIC values (μM) obtained for active antimicrobials from Library 1, plus one randomly selected inactive compound (**47**) and **AT**. Colour of fold change indicates the shift of the inverted protocol compared to the conventional protocol.*

		ATCC 9144			NCTC 13616		
		Inverted	Conventional	Fold	Inverted	Conventional	Fold
Compound	**6**	125	62.5	2	31.25	31.25	1
	**8**	31.25	500	16	7.8125	500	64
	**27**	62.5	125	2	31.25	62.5	2
	**28**	125	62.5	2	31.25	31.25	1
	**29**	31.25	62.5	2	62.5	31.25	2
	**47**	>1000	>1000	-	>1000	>1000	-
	**94**	250	250	1	125	125	1
	**AT**	31.25	15.625	2	31.25	15.625	2
Increase				Decrease			

We also benchmarked the assays against the known antibacterial agents, ciprofloxacin and cefoxitin. The MIC values for the known agents are shown in Table S5, and demonstrate that (i) the NCTC 12923 (Gram-negative) strain is not unusually resistant to antimicrobial action, and (ii) that ATCC 13616 is indeed MRSA while NCTC 9144 is MSSA.

Table S5 Comparable modal MIC values (μM) obtained for the known antimicrobial agents ciprofloxacin and cefoxitin, using the conventional MIC protocol. Values shown in red are classed as resistant according to European Committee on Antimicrobial Susceptibility Testing (EUCAST) breakpoints.

**Strain**	**Known antimicrobial agent**	
	Ciprofloxacin	Cefoxitin
ATCC 9144	1	1
NCTC 13616	64–128	32
NCTC 12923	0.008	n/a

S4.4 High throughput bacterial screen

The high throughput bacterial screen was completed with the same method as the inverted MIC protocol. A polypropylene compound plate (Greiner 655201) was prepared by diluting SSA in 100% DMSO, while a polystyrene assay plate (Greiner 655182) was inoculated with 0.011 OD of the required bacteria in TSB. SSA aliquots of 10 µl were added to the assay plate before the addition of Rezasurin (Thermofischer, UK). Completed assay plates were incubated at 37°C in a Clariostar Plus plate reader. The Optical density and fluorescence intensity were measured after 5 hours and 20 hours of incubation. The results are show in Figure S 19.

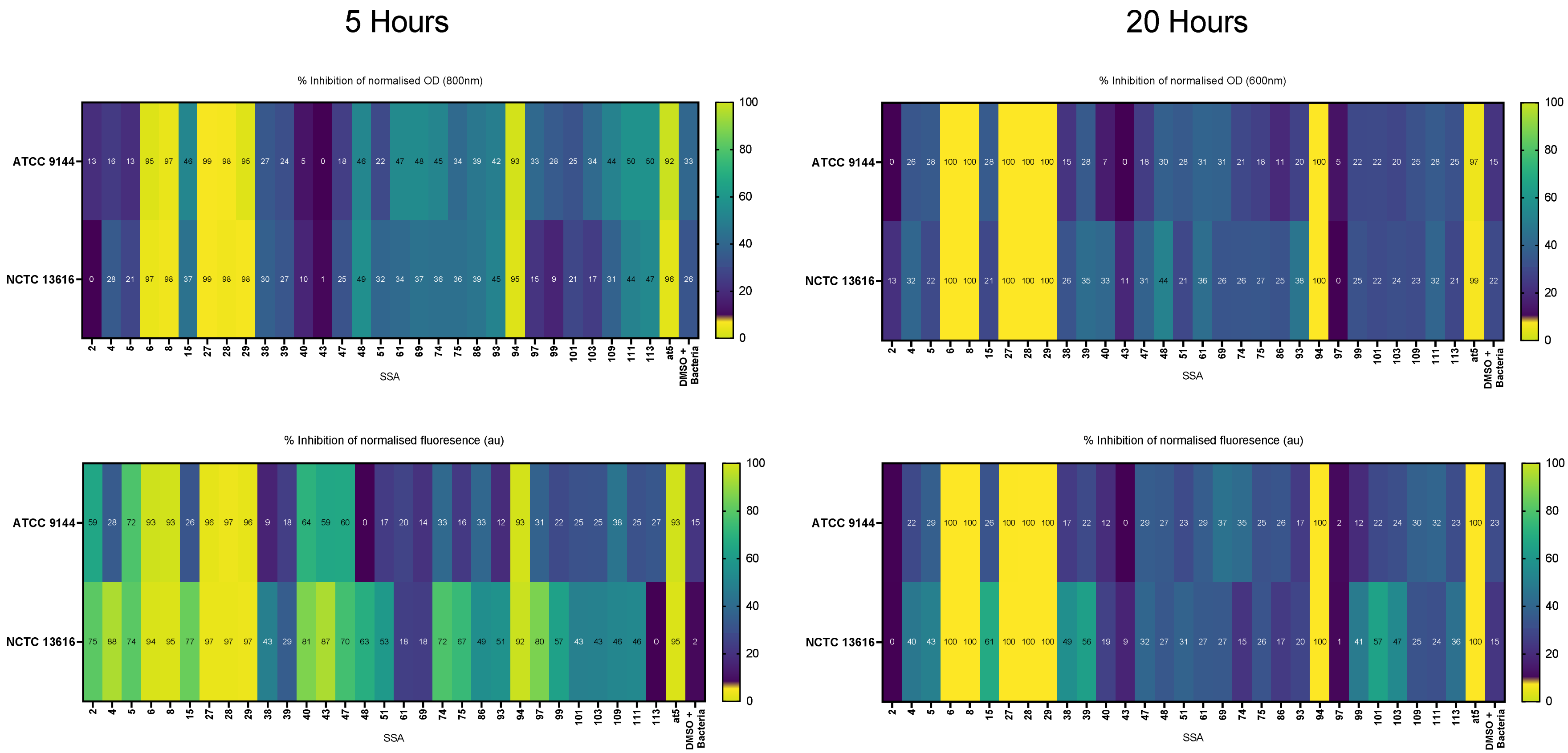

Figure S 19 Heatmaps detailing the percentage of antimicrobial growth inhibition obtained for ***Library 1*** *and* ***AT*** *at 100 µM against S. aureus ATCC 9144 and S. aureus NCTC 13616 at 20 hours. Heatmaps display the percent inhibition of either optical density (turbidity or growth), and inhibition of fluorescence (metabolic activity) at 20 hours. All values were blank adjusted and normalised to the positive control for complete inhibition (DMSO + broth, to calibrate 100% inhibition of growth/ metabolic activity).*

S4.5 Investigating the impact of chloride anions

Overnights of *S. aureus* NCTC 13616 were prepared in TSB, following incubation 1 mL aliquots were centrifuged at 12,000 rpm for 2 minutes, the supernatant was discarded, and pellets were resuspended in either HBSS or gluconate buffer. Aliquots were centrifuged under the same conditions again, the supernatant was discarded, and pellets were resuspended in the appropriate buffer. OD600 was measured and 0.1 OD inoculum were prepared of each strain in each buffer. SSAs were diluted in 100% DMSO in drug plates, and 190 µL of inoculum was added to all wells of the assay plate. From the SSA drug plate, 10 µL of the desired compound was aspirated, and this was dispensed into the appropriate well of the assay plate. Assay plates were incubated for 18 hours at 37℃ before the OD600 was measured in a Clariostar plate reader. Finally, 10 µL of Resazurin was added to each well. The fluorescence (excitation = 544 nm, emission = 599 nm) of these plates was measured immediately, then incubated for 1 hour and 45 minutes before reading again. A reduction in normalised fluorescence below 10% of the positive control was interpreted as inhibition of cell viability and an indication of compound activity. Modal concentrations were determined where possible and ranges are reported where not determined, results are reported in Table S6.

*Table S6 Comparable modal MIC values (µM) from Resazurin assays for active compounds in Library 1, plus one randomly selected inactive compound (**47**) and **AT** in HBSS and gluconate buffers. Reported values are for S. aureus NCTC 13616.*

		Mode	
		HBSS	Gluconate
Compound	**6**	7.81 - 31.2	7.81 - 31.2
	**8**	250	125
	**27**	3.91 - 15.6	3.91 - 31.2
	**28**	7.81 - 31.2	7.81 - 31.2
	**29**	3.91 - 31.2	3.91 - 31.2
	**47**	≥1000	≥1000
	**94**	31.25	31.25
	**AT**	7.81	7.81

S5 Dynamic light scattering

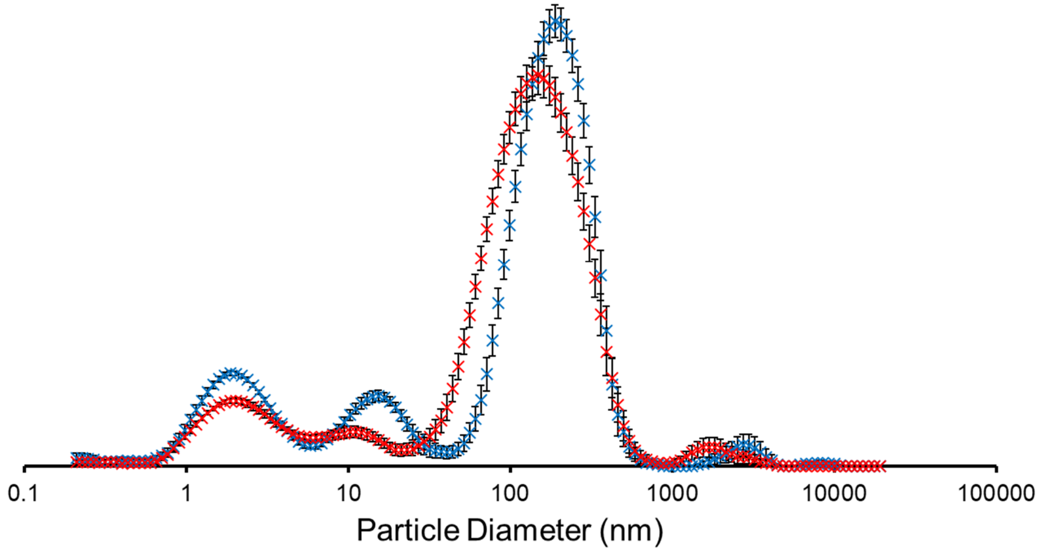

*Figure S 20 An overlay of average intensity particle size distributions calculated using 10 DLS runs of **6** (0.1 mM) in TSB/5.0 % DMSO (red) and TSB/5.0 % DMSO (blue) at 298 K.*

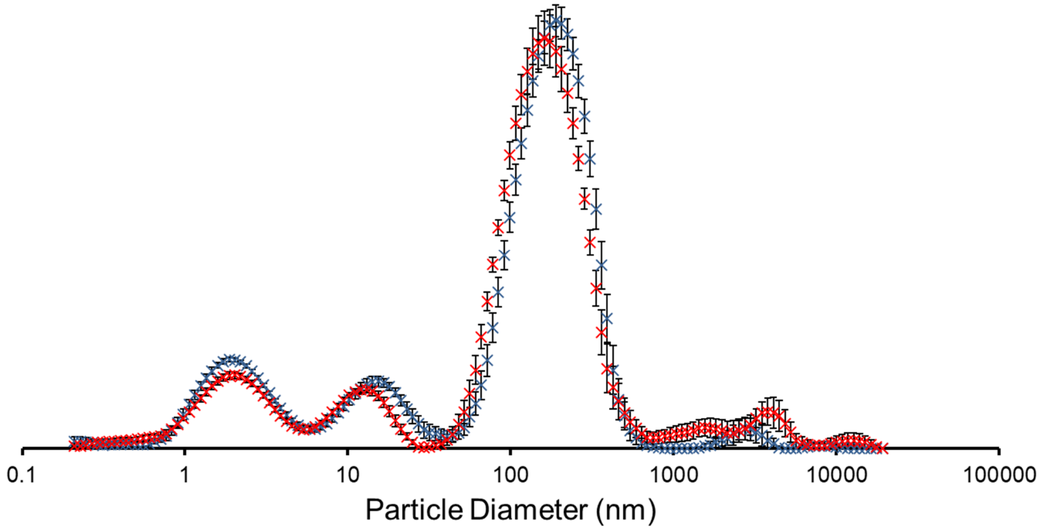

*Figure S 21 An overlay of average intensity particle size distributions calculated using 10 DLS runs of **8** (0.1 mM) in TSB/5.0 % DMSO (red) and TSB/5.0 % DMSO (blue) at 298 K.*

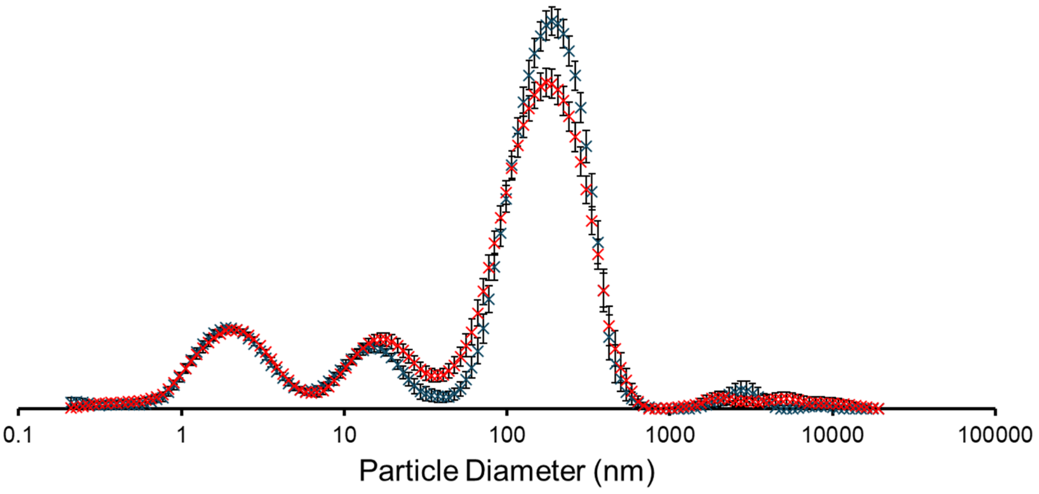

*Figure S 22 An overlay of average intensity particle size distributions calculated using 10 DLS runs of **27** (0.1 mM) in TSB/5.0 % DMSO (red) and TSB/5.0 % DMSO (blue) at 298 K.*

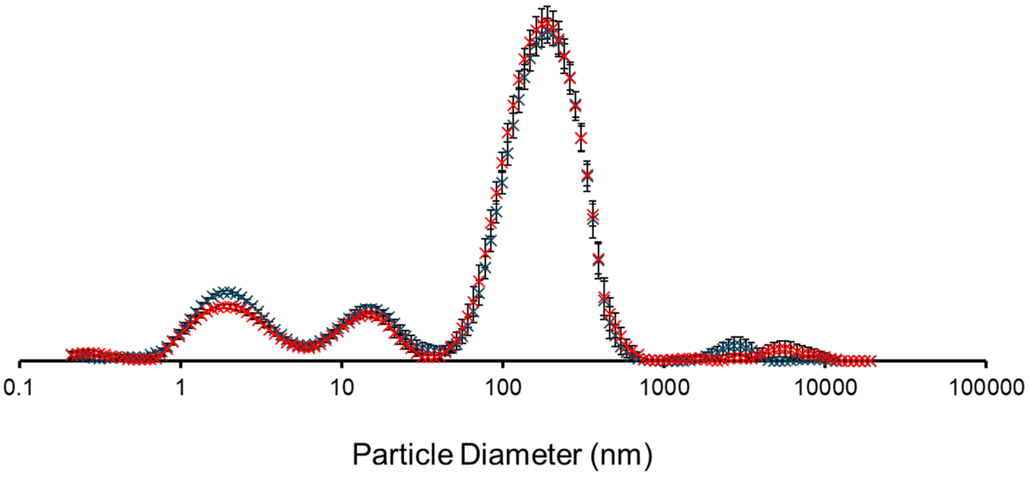

*Figure S 23 An overlay of average intensity particle size distributions calculated using 10 DLS runs of **28** (0.1 mM) in TSB/5.0 % DMSO (red) and TSB/5.0 % DMSO (blue) at 298 K.*

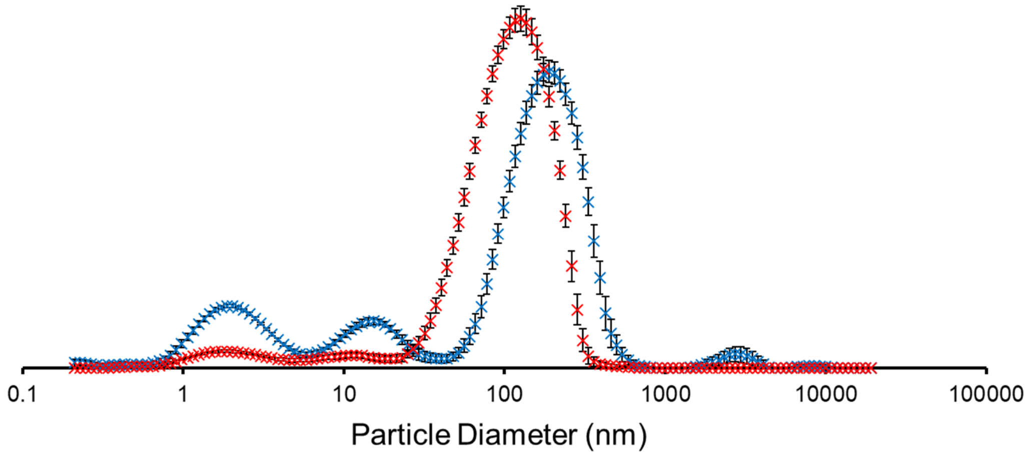

*Figure S 24 An overlay of average intensity particle size distributions calculated using 10 DLS runs of **29** (0.1 mM) in TSB/5.0 % DMSO (red) and TSB/5.0 % DMSO (blue) at 298 K.*

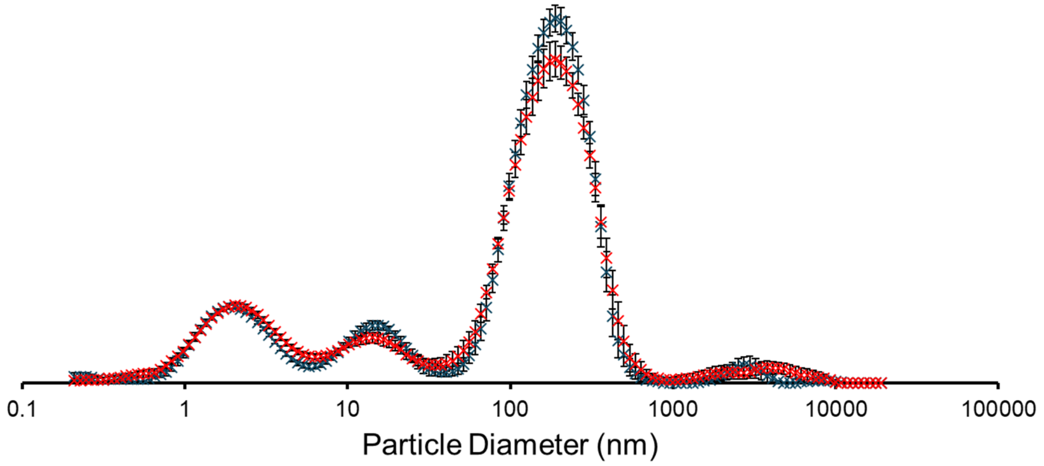

*Figure S 25 An overlay of average intensity particle size distributions calculated using 10 DLS runs of **94** (0.1 mM) in TSB/5.0 % DMSO (red) and TSB/5.0 % DMSO (blue) at 298 K.*

S6 Lipid extraction and NMR Quantification

Tryptone Soy Broth (TSB, Merck, UK) was prepared following manufacturer's instructions and sterilised by autoclaving for 15 minutes at 121°C. Per strain, two overnight cultures were prepared by inoculating 3 mL of TSB with the appropriate strain and incubated at 200 rpm and 37°C. Each overnight culture was added to 500 mL of TSB in 1000 mL conical flasks, and these were incubated overnight at 200 rpm and 37°C. Cells were harvested by centrifugation at 7,500 rpm for 10 min at room temperature. Supernatants were decanted, leaving approximately 2 mL of residual medium in each vessel. Pellets were resuspended by vortexing

and combined into 15 mL conical tubes, ensuring that the volume in each tube did not exceed 5 mL.

The resuspended pellets were boiled at 98°C for 25 min. Samples were then snap-frozen in a dry ice–ethanol bath and stored at –80°C for analysis.

The cells were then defrosted and resuspended before being sonicated to disrupt the cellular membranes, sonication consisted of max power sonication with 50% amplification for 30 seconds on, 30 seconds off cycles for a total sonication time of 15 minutes.

After sonication the lipids were harvested via centrifugation at 70,000 RPM for 45 minutes at 4°C. The supernatant was again discarded, and the pellet was resuspended in 3 mL of water. The resuspended pellet was then homogenised using a glass Dounce homogeniser to create a homogenous suspension.

The homogenised solution was added to a glass vial before the addition of 15 mL of chloroform and 7.5 mL of methanol. The mixture was stirred for 1 hour before the addition of 6.25 mL of water, the mixture was then left to phase separate until there were three distinct phases:

- The top layer of methanol containing parts of the cell which are soluble in methanol
- A middle layer containing parts which are soluble in neither chloroform nor methanol
- The bottom layer of chloroform containing soluble lipids.

The chloroform layer containing the soluble lipids was removed and the chloroform was evaporated off using a stream of nitrogen gas. The lipids were then resuspended in 75% deuterated chloroform, 24.95% deuterated methanol and 0.05% tetramethylsilane (TMS).

^{1}H–^{31}P HSQC spectra were collected on the extracted lipids using a Bruker Avance III spectrometer at a proton frequency of 600 MHz at 298 K. This spectrometer is equipped with a QCIP cryoprobe with a standard ^{31}P pre-amplifier without enhanced sensitivity from cryogenic cooling. The collected spectra were then analysed using MestReNova software.[12]

S7 Automated data processing

One limitation on throughput in this workflow is the time required to process the data and fit EC_{50} values. To minimise manual handing and standardise all outputs for analysis, we implemented an automated pipeline that (i) links plate reader files to experimental metadata and (ii) converts raw CSV exports into structured tables for downstream fitting.

First, we defined a standardised naming format so each plate reader output can be linked to the corresponding metadata. Second, we built an inline user interface (UI) in a Jupyter notebook (Figure S26) that allows the user to select a predefined plate layout, and enter or edit metadata linked to plate cells, without interacting with the underlying code. The resultant plate metadata is then shown to the user for review (examples in Figure S27).

Once confirmed, the notebook then reads the plate reader CSV sequentially, extracting the rows corresponding to the relevant measurement wavelengths, and assembles theses into pandas DataFrames for subsequent processing and EC_{50} fitting.

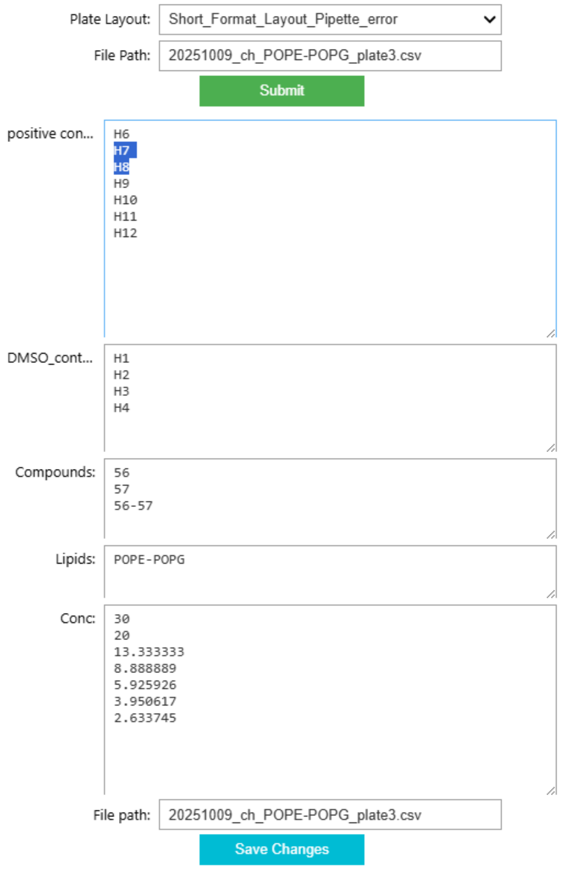

Figure S 26 The notebook UI for metadata input and modification.

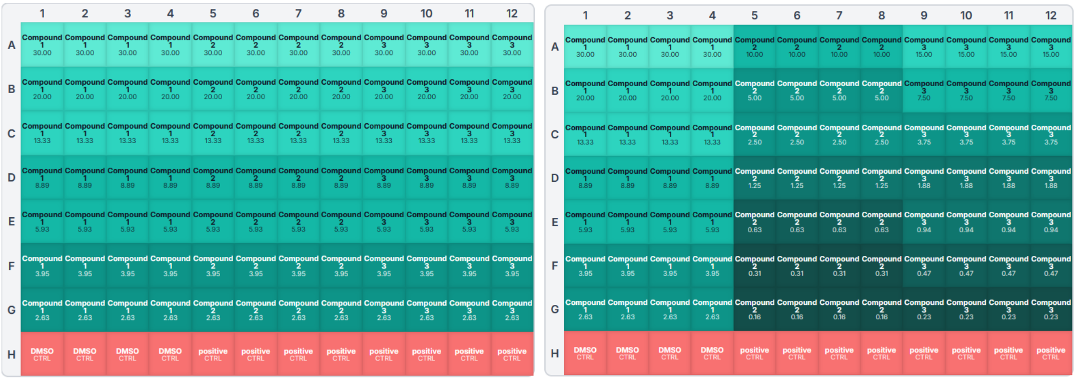

Figure S 27 Example plate layouts used in the processing workflow. Left: standard plate layout with fixed concentrations. Right: standard plate layout with variable concentrations

Once selected the metadata is displayed within the UI shown in Figure S27. The average positive control is selected within the UI and therefore can be modified to remove cells with experimental error. Along with this DMSO control, compounds and concentrations can be updated across the panels rather than having to be edited for individual cells.

The data are converted to a ratiometric readout (460 nm / 403 nm), then min–max normalised using the positive and negative control values defined in the metadata, using the following equation:

$$percentage\_change\_df = \frac{\left(\frac{df_{460}}{df_{403}} - avg\_dmso\right) \times 100}{avg\_pos - avg\_dmso}$$

The percentage change across the plate is then displayed in another UI within the next cell of the Jupyter notebook, where the user can remove or modify if any cells which are outliers due to experimental error or artefacts (Figure S28). For traceability, the original value and well location are stored in the workflow output (in the “all data” column). Concentrations, compounds and cell lines can also modified on a per cell basis rather than as a batch wide update.

Interactive Data Explorer

Plate %Change

key	1	2	3	4	5	6	7	8	9	10	11	12
A	56.1	49.2	41.1	41.2	67.6	67.7	54.3	53.8	40.3	39.9	46.1	47.5
B	7.6	7.1	10.0	10.2	22.3	24.8	25.7	26.3	17.5	17.2	16.5	16.6
C	4.4	4.3	6.6	6.2	12.5	13.6	13.6	13.5	9.9	9.9	9.5	9.8
D	3.1	2.9	3.8	4.0	7.9	7.9	8.2	8.4	6.1	6.2	5.7	5.7
E	2.0	2.0	2.5	2.6	4.6	4.1	4.6	4.4	3.5	3.5	3.4	3.7
F	1.2	1.5	1.6	1.7	2.6	2.7	2.3	2.6	1.7	2.4	2.2	2.4
G	1.0	1.1	1.2	1.0	1.8	1.6	2.0	2.1	1.8	1.5	1.4	2.7
H	0.1	0.1	-0.1	-0.1	nan	94.9	nan	2.2	100.6	100.1	102.0	102.4

Concentration

key	1	2	3	4	5	6	7	8	9	10	11	12
A	30	30	30	30	30	30	30	30	30	30	30	30
B	20	20	20	20	20	20	20	20	20	20	20	20
C	13.333333	13.333333	13.333333	13.333333	13.333333	13.333333	13.333333	13.333333	13.333333	13.333333	13.333333	13.333333
D	8.888889	8.888889	8.888889	8.888889	8.888889	8.888889	8.888889	8.888889	8.888889	8.888889	8.888889	8.888889
E	5.925926	5.925926	5.925926	5.925926	5.925926	5.925926	5.925926	5.925926	5.925926	5.925926	5.925926	5.925926
F	3.950617	3.950617	3.950617	3.950617	3.950617	3.950617	3.950617	3.950617	3.950617	3.950617	3.950617	3.950617
G	2.633745	2.633745	2.633745	2.633745	2.633745	2.633745	2.633745	2.633745	2.633745	2.633745	2.633745	2.633745
H												

S 28 the UI to update Concentrations, compounds and cell lines and percentage change.

Once confirmed, these data are grouped by compound, vesicle type and then concentration within the Python code, with an average taken for each distinct grouping. This is then stored as mean response, standard deviation between the responses list to allow for manual processing in Origin, or use in plotting via Python or machine learning analysis on a large subset of data. These lists are stored in the same row as other metadata the complete list is as follows. The full list of column titles includes Date, Comp1, Comp2, Ratio, Units of conc, DMSO Control (negative control), Positive Control, Raw Data (a python dictionary containing all the data used within python processing), Response (% efflux in this case), Concentration, STD, Rep, Initials, Lipid_Type, and Plate_ID.

For this work we have data processed and summarised using the workflow into a summary_results.csv. From here we have fit and plot the data using Python to replicate within error the EC_{50} value generated through origin. The plots generated are shown in Figure S11-14 (by comparison to those generated in Origin) for visual inspection and a comparison of EC_{50} values are shown in Table S7.

Table S7 a comparison of the EC50 values generated from our Python workflow with the resultant summary data fitted to the Hill1 formula in Python, by comparison to the EC_{50} values generated from Excel data processing and followed by fitting to the Hill1 equation in Origin 2019.

**Comp1**	**Comp2**	**Lipid Type**	**EC50 (Python fitting)**	**EC_{50} (Origin)**
73		POPC	4.42	4.37
73		POPG	11.18	11.16
73		POPE-POPG	8.31	8.32
72	73	POPC	7.91	7.81
72	73	POPG	10.95	10.81
72	73	POPE-POPG	11.39	11.27
72		POPC	33.32	32.92
72		POPG	20.03	20.03
72		POPE-POPG	35.20	34.87
57		POPC	48.95	49.04
57		POPG	16.87	16.71
57		POPE-POPG	36.49	36.15
56	57	POPC	69.81	69.14
56	57	POPG	18.36	18.32
56	57	POPE-POPG	52.15	51.66
56		POPC	90.00	96.11
56		POPG	30.61	30.48
56		POPE-POPG	61.65	60.22

In addition to efficiently reproducing the EC_{50} values and dose–response plots, the standardised approach to processing and formatting enables downstream computational analyses, such as QSAR modelling across large libraries of EC_{50} values generated using this pipeline.

The script is available with all raw, manually processed and summary data generated from the workflow at https://github.com/ta1u18/EC50-high-throughput-workflow.

S8 References

1. K. Yang, L. C. Lee, H. A. Kotak, E. R. Morton, S. M. Chee, D. P. M. Nguyen, A. Keskküla and C. J. E. Haynes, *Chemistry–Methods*, 2025, **n**/**a**, e202400084.
2. L. J. White, S. N. Tyuleva, B. Wilson, H. J. Shepherd, K. K. L. Ng, S. J. Holder, E. R. Clark and J. R. Hiscock, *Chem. Eur. J.*, 2018, **24**, 7761-7773.
3. L. R. Blackholly, H. J. Shepherd and J. R. Hiscock, *CrystEngComm*, 2016, **18**, 7021-7028.
4. L. J. White, N. J. Wells, L. R. Blackholly, H. J. Shepherd, B. Wilson, G. P. Bustone, T. J. Runacres and J. R. Hiscock, *Chem. Sci.*, 2017, **8**, 7620-7630.
5. N. Allen, L. J. White, J. E. Boles, G. T. Williams, D. F. Chu, R. J. Ellaby, H. J. Shepherd, K. K. L. Ng, L. R. Blackholly, B. Wilson, D. P. Mulvihill and J. R. Hiscock, *ChemMedChem*, 2020, **15**, 2193-2205.
6. A. Rutkauskaite, L. J. White, K. L. F. Hilton, G. Picci, L. Croucher, C. Caltagirone and J. R. Hiscock, *Org. Biomol. Chem.*, 2022, **20**, 5999-6006.
7. J. E. Boles, C. Bennett, J. Baker, K. L. F. Hilton, H. A. Kotak, E. R. Clark, Y. Long, L. J. White, H. Y. Lai, C. K. Hind, J. M. Sutton, M. D. Garrett, A. Cheasty, J. L. Ortega-Roldan, M. Charles, C. J. E. Haynes and J. R. Hiscock, *Chem. Sci.*, 2022, **13**, 9761-9773.
8. P. I. A. Poopla, T. Allam, R. Lilley, C. Manwani, O. Keers, J. Tan, K. Yang, Y. Long, L. J. White, K. H. L. F. Hilton, J. Rankin, J. Baker, C. Bennet, H. Wilson, E. R. Morton, A. Keskkula, J. M. Sutton, C. K. Hind, M. D. Garrett, C. J. E. Haynes and J. R. Hiscock, *ChemRxiv*, 2025, DOI: 10.26434/chemrxiv-2025-bjgl9.
9. A. Rutkauskaite, L. J. White, J. E. Boles, K. L. F. Hilton, M. Clifford, B. Patenall, B. R. Streather, D. P. Mulvihill, S. A. Henry, M. Shepherd, J. M. Sutton, C. K. Hind and J. R. Hiscock, *Supramol. Chem.*, 2021, **33**, 677-686.
10. K. L. F. Hilton, H. J. F. Steyn, K. S. Luthuli, M. Rice, B. R. Streather, E. Sweeney, L. J. White, F. R. Morgan, J. Rankin, J. Baker, C. Bennett, H. B. Wilson, P. A. Hailey, M. D. Garrett, J. L. Ortega-Roldan, J. M. Sutton, C. K. Hind, C. H. Pohl and J. R. Hiscock, *Journal of Materials Chemistry B*, 2025, **13**, 8239-8251.
11. L. J. White, B. Streather, J. M. Sutton, J. Rankin, J. Baker, C. Bennett, H. B. Wilson, C. K. Hind and J. R. Hiscock, *Org. Biomol. Chem.*, 2025, DOI: 10.1039/D5OB01615K.
12. E. Medina-Carmona, L. Varela, A. C. Hendry, G. S. Thompson, L. J. White, J. E. Boles, J. R. Hiscock and J. L. Ortega-Roldan, *Chem. Commun.*, 2020, **56**, 11665-11668.
